# Cross-feeding enables robust coexistence between four bacterial species

**DOI:** 10.1073/pnas.2607664123

**Published:** 2026-09-17

**Authors:** Snorre Sulheim, Miguel Teixeira, Eric Ulrich, Alisson Gillon, Samuele E. A. Testa, Prajwal Padmanabha, Daniel Machado, Sara Mitri

**Affiliations:** ^a^ Department of Fundamental Microbiology, University of Lausanne, Lausanne 1015, Switzerland; ^b^ Department of Biotechnology and Food Science, Norwegian University of Science and Technology, Trondheim 7491, Norway

**Keywords:** coexistence, cross-feeding, synthetic communities, microbial interactions, consumer–resource modeling

## Abstract

Resource supply is generally considered a dominant driver of microbial diversity. Here, using a four-species bacterial consortium, we asked whether coexistence can emerge from interactions between species rather than being dictated by the external environment. Across 31 simple nutrient conditions, including 16 single-resource environments, all four species survived and coexisted over eight growth-and-dilution cycles. We then chose 28 additional conditions to further probe the boundaries of coexistence by varying resource concentrations, temporal dynamics, nutrient complexity and relief of auxotrophy-driven dependencies, and only observed the extinction of a single species in one of these conditions. Although the community composition in each environment was largely shaped by species’ fitness on the supplied resources, experimental assays and consumer-resource modeling showed that the coexistence was explained by cross-feeding and niche partitioning of metabolic byproducts. These metabolic interactions were strong enough to sustain coexistence even for species that failed to exhibit significant growth on the supplied resources in monoculture. Furthermore, robust coexistence across environments appears to be an emergent property of the community itself, ingrained in the members’ metabolic byproduct profiles and niche differences. These findings demonstrate that the richness of microbial consortia is ultimately governed by these intrinsic metabolic traits, rather than being strictly dictated by the number or identity of the externally supplied resource.

## Significance

Understanding the drivers of microbial diversity is essential for managing natural ecosystems and designing synthetic microbiomes. This study tests the relative importance of resource supply vs. species attributes, demonstrating that a four-species consortium can coexist across 31 chemically and metabolically diverse one- and two-carbon source environments. By systematically testing and ruling out alternative stabilizing mechanisms, we show that coexistence is an emergent property of the consortium, sustained by metabolic cross-feeding and niche partitioning. Guided by computational models, we identify hallmarks of robust coexistence in simple environments, including high variance in resource affinities and growth on partner-derived metabolites. Our work demonstrates how microbes modify their environment to sustain high diversity and provides principles for designing synthetic microbiomes that persist across environments.

The stunning microbial diversity found in natural environments has impressed microbiologists since the 17th century (1–5). This diversity—as with nonmicrobial ecosystems (6, 7)—correlates positively with the stability and functions of microbial ecosystems (8). In the human gut, for instance, diminished microbiome diversity is associated with recurrent *Clostridium difficile* infections (9), while in terrestrial systems, soil microbiome diversity drives plant productivity and nutrient retention (10). Understanding when and why microbes coexist is therefore critical for managing ecosystem stability and function.

Theoretical expectations of diversity are governed by the competitive exclusion principle, which posits that the number of coexisting species in a well-mixed environment cannot exceed the number of limiting resources (11, 12). Historically, the external resource supply was expected to strictly limit the available ecological niches. However, empirical observations from nature and laboratory experiments frequently reveal richness that exceeds these theoretical bounds (13–18). An updated understanding is that microbial cross-feeding is common (19): By releasing a large diversity of metabolites into the surrounding environment (20–22), microbes actively increase the number of resources beyond the external supply, allowing tens of species to coexist on even a single provided carbon source (17, 18, 23).

Several additional mechanisms can increase diversity, including environmental fluctuations combined with variation in growth traits, favoring different species at different times (15, 24), predation or inhibition of the dominant species (25, 26), and cross-detoxification (27, 28). Central to most of these mechanisms is negative frequency dependence, whereby rare genotypes have a growth advantage over the more common ones, leading to stable equilibrium frequencies.

Despite these insights, it remains challenging to predict when species will coexist or not (29, 30). A key knowledge gap is the incomplete understanding of the relative importance of the identity and concentration of supplied resources, microbial traits, and interspecies interactions in determining coexistence outcomes. Furthermore, to confidently distinguish extinction from low-abundance survival, individual community members must be quantifiable down to very low frequencies. Here, we bridge this gap by profiling a synthetic four-species bacterial community across an extensive matrix of resource supply conditions, using selective plating to track individual species abundances across eight orders of magnitude for eight growth-and-dilution cycles. We demonstrate that these taxonomically diverse bacteria coexist consistently—with only a single exception—across simple environments supplied with only one or two carbon sources. We rule out alternative stabilizing factors as explanations for the observed coexistence, including auxotrophy-driven dependencies and resource fluctuations. Rather, and in line with previous observations (17, 18, 31), we find that microbes actively increase the chemical complexity of their environment through niche construction, maintaining coexistence via cross-feeding. By additionally simulating thousands of communities using a mathematical model, we conclude that coexistence emerges as a property of the community itself, shaped by cross-feeding patterns and niche differences, rather than by the concentration or identity of the externally supplied resources.

## A Synthetic Four-Species Community Robustly Coexists Across Simple Nutrient Environments.

To understand how coexistence depends on supplied resources, we used a synthetic model community comprising four bacterial species: *Agrobacterium tumefaciens* (At), *Comamonas testosteroni* (Ct), *Microbacterium liquefaciens* (Ml), and *Ochrobactrum anthropi* (Oa). These strains were originally isolated from toxic metal-working fluids (32), and have since been used as a model system to study synthetic communities (27, 28, 33). A critical advantage of this consortium is the ability to resolve individual species abundances on selective agar media even at low frequencies (28), essential for distinguishing between competitive exclusion and species declining to a low-abundance steady-state. These species possess varying metabolic capabilities and nutritional dependencies (34, 35). Notably, At and Ct are prototrophs while previous experiments have shown that Oa and Ml depend on cocultivated partners for prolonged growth (28). To characterize these dependencies, we first identified putative auxotrophies based on the absence of essential biosynthetic enzymes in the annotated proteomes, and then conducted drop-out or drop-in assays to confirm or reject these metabolic dependencies. Gaps in vitamin biosynthesis pathways matched in vitro experiments, verifying that Oa is a thiamine auxotroph and Ml is auxotrophic for both biotin and thiamine (*SI Appendix*, Fig. S1 *A*–*C*). In contrast, predicted amino acid auxotrophies of Ml did not match in vitro results, which revealed an absolute dependency on exogenous cysteine. Furthermore, while Ml is genetically prototrophic for proline, supplementation was required to bypass a substantial 48-h lag phase (*SI Appendix*, Fig. S1 *D* and *E*). By incorporating these natural auxotrophies, this four-species community serves as a tractable model community that captures the functional dependencies found in natural microbiomes (36).

We characterized the growth of each species across 33 metabolically and chemically diverse carbon sources (*SI Appendix*, Fig. S2 *A* and *B*), revealing distinct niche differences and a trade-off between versatility and growth rate (*SI Appendix*, Fig. S2 *C* and *D*). Ct was the least versatile, unable to utilize 14/33 substrates (including all sugars), but frequently exhibited the fastest growth on organic acids and amino acids. In contrast, Ml utilized 32 of 33 tested carbon sources but grew slowly in most conditions. From this set, we selected 16 carbon sources to construct 32 resource environments for our initial community assembly experiment, consisting of 16 single-resource and 15 paired-resource, and a no-carbon control condition. These substrates included organic acids, sugars, amino acids, an alcohol (glycerol), a sugar-alcohol (myo-inositol), and a nucleoside (inosine), and all environments were normalized to an equal amount of carbon (90 mM C). The community was then subjected to eight growth-and-dilution cycles, each lasting 72 h in these different media. By selecting a chemically and metabolically diverse array of carbon sources, as well as choosing carbon sources favoring different species, we sought to maximize environmental variability and test the boundaries of coexistence.

**Fig. 1.**
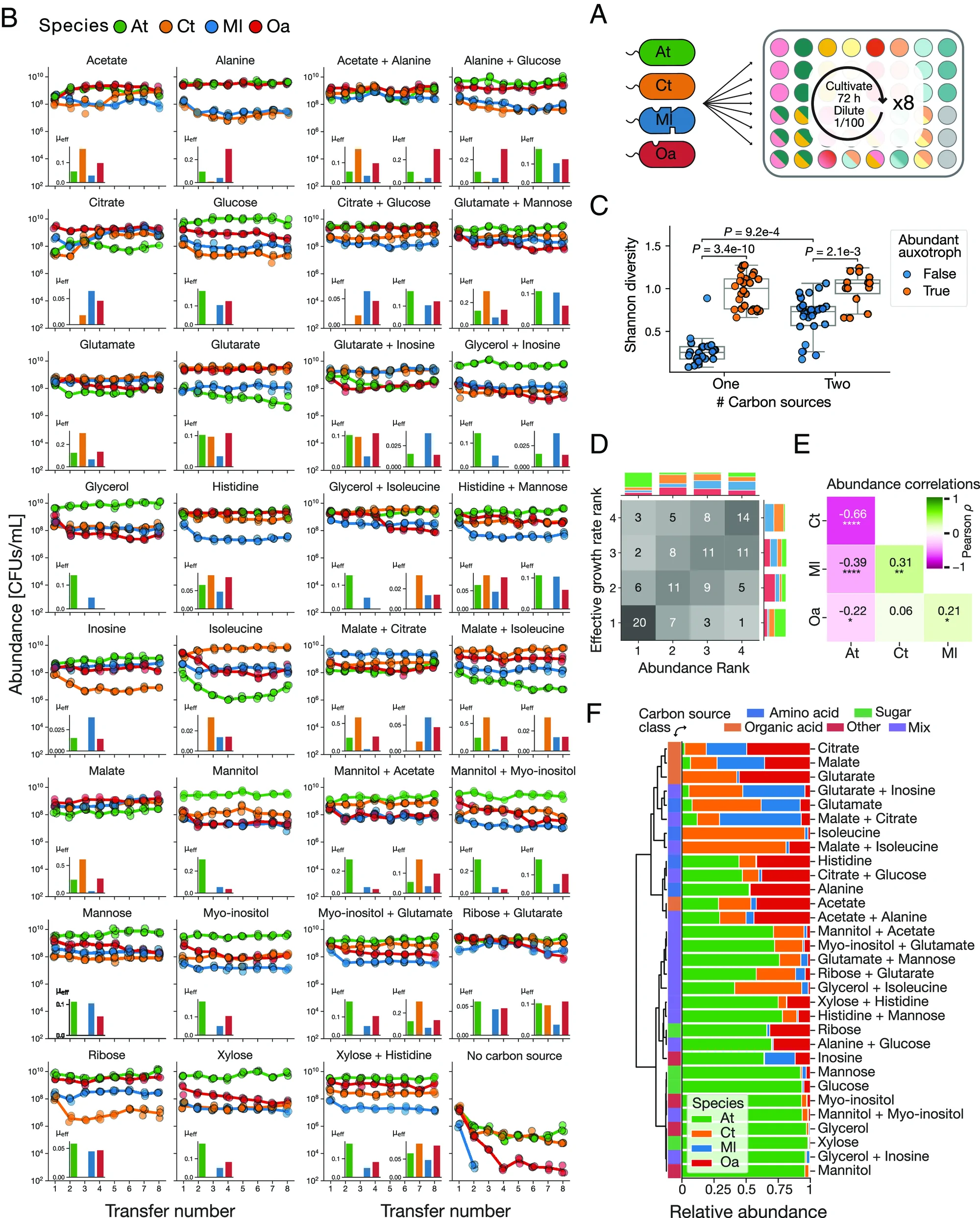
Four species coexist across 31 conditions. (*A*) Schematic illustration of the community assembly experiments. (*B*) Quantified species abundance at the end of each of the 8 consecutive batch transfers. Single carbon source environments are shown in the two leftmost columns, while environments with two carbon sources and the no-carbon control are shown in the two rightmost columns. Inset bar charts show the effective growth rates for each species measured in separate monoculture experiments; for the two-carbon source environments, left and right insets correspond to the first and second carbon sources, respectively. (*C*) Environments with an abundant auxotroph (Oa or Ml is >25% of the total population) have increased diversity (*SI Appendix*, Fig. S3*B*), but this increase is only significant for the single carbon source environments. For the environments without an abundant auxotroph, the diversity is significantly higher when two carbon sources are provided. Statistical tests: Kruskal–Wallis followed by Dunn’s post hoc test with Benjamini–Hochberg correction. (*D*) Community assembly abundance ranks are highly predictable from monoculture effective growth rates, with 84.7% (105/124) of observations falling within ±1 of the predicted rank. (*E*) Pearson correlations of species’ abundance in the community assembly experiment (*P < 0.05, **P < 0.01, ***P < 0.001, and ****P < 1e-4). (*F*) Final relative abundance in the community assembly experiments (mean of three replicates over the last two time points clustered by similarity in community composition).

Given the limited number and diversity of provided carbon sources, and previous work (37, 38), our expectation was that competitive exclusion would be prevalent. Instead, all four species coexisted in every environment tested, reaching stable steady-states by the eighth transfer and often already after four or five transfers (Fig. 1 *A* and *B*). Even species unable to utilize the provided carbon sources were able to reach steady-state population sizes, in some environments up to 10^8^ CFUs/mL, suggesting that whatever allows these species to grow is sufficient to sustain such population sizes and an average growth rate above the ∼0.064 h^−1^ effective dilution rate imposed by the 1/100 dilution every 72 h (citrate and inosine environments diluted 1/10, see *Materials and Methods*). All species except Ml were also still present at the end of the experiment in the no-carbon control, although at significantly lower abundances than in the other environments (P < 1e-3 for all species and conditions, see *Materials and Methods*). Low-level growth in no-carbon conditions is a well-known challenge in microbial cultures that has been shown to support low-abundance communities in similar experiments previously (18).

Despite observing coexistence in all environments, we still expected environments containing two carbon sources instead of one to increase evenness by introducing an additional primary niche. However, Shannon diversity was not primarily driven by the number of supplied resources (*P*= 0.21, Mann–Whitney U; *n*_1_= 48, *n*_2_= 45, *SI Appendix*, Fig. S3*A*). Instead, the prevalence of auxotrophic members emerged as the principal determinant of community structure. Environments where an auxotroph (Oa and Ml) constituted more than 25% of the total population showed a significantly higher diversity (*P*= 7.1e-11, Mann–Whitney U, *SI Appendix*, Fig. S3*B*). By partitioning the data by both resource complexity and auxotroph prevalence, we observed that auxotroph prevalence had the strongest influence on single-carbon environments (Fig. 1*C*). In the absence of an abundant auxotroph, increased resource complexity did increase Shannon diversity (Fig. 1*C*), but this effect was secondary to the stabilizing influence of metabolic dependency. This phenomenon is likely driven by the inherent constraints of cross-feeding: The reliance of auxotrophs on partners for essential vitamins or amino acids effectively caps their relative abundance, often resulting in two or more species persisting at nearly equal frequencies even on a single substrate (see e.g., alanine or glutarate environments, Fig. 1*B*).

We next investigated the extent to which species’ rank abundances in the assembled communities could be predicted from their growth in monoculture. Monoculture growth rate ranks were significantly correlated with final abundance ranks (Kendall’s τ=0.33, *P*= 1.3e-5, N=124), but predictive accuracy improved when using “effective” growth rates that also accounted for lag and yield differences (Kendall’s τ=0.46, *P*= 1.2e-9, N=124, Fig. 1*D*, see *Materials and Methods*). The most abundant species was predicted with higher fidelity than subordinate members, likely reflecting that while primary resource utilization governs dominance, the relative abundances of subordinate species are also shaped by secondary processes, such as metabolite cross-feeding, that cannot be informed by monoculture growth data. To interrogate these putative interactions, we analyzed species abundance correlations, recognizing that such associations integrate the net effects of both nutrient competition and cross-feeding. While At was negatively correlated with all other community members, Ml’s abundance was positively correlated with Oa and Ct (Fig. 1*E*). The negative correlations involving At reflect its competitive dominance in the environments where it exhibits the highest effective growth rate (sugars). In these conditions, it frequently exceeds more than 90% of the population, suggesting that it provides little support for cocultured partners (Fig. 1*F*). In contrast, the positive correlations in abundances of Ml with Ct and Oa were not mirrored by correlations in effective growth rate (*SI Appendix*, Fig. S3*D*). This decoupling suggests their positive association is not driven by shared resource preferences, but is rather an emergent result of mutualistic or commensal cross-feeding interactions.

## Coexistence Is Robust to Environmental Perturbations.

**Fig. 2.**
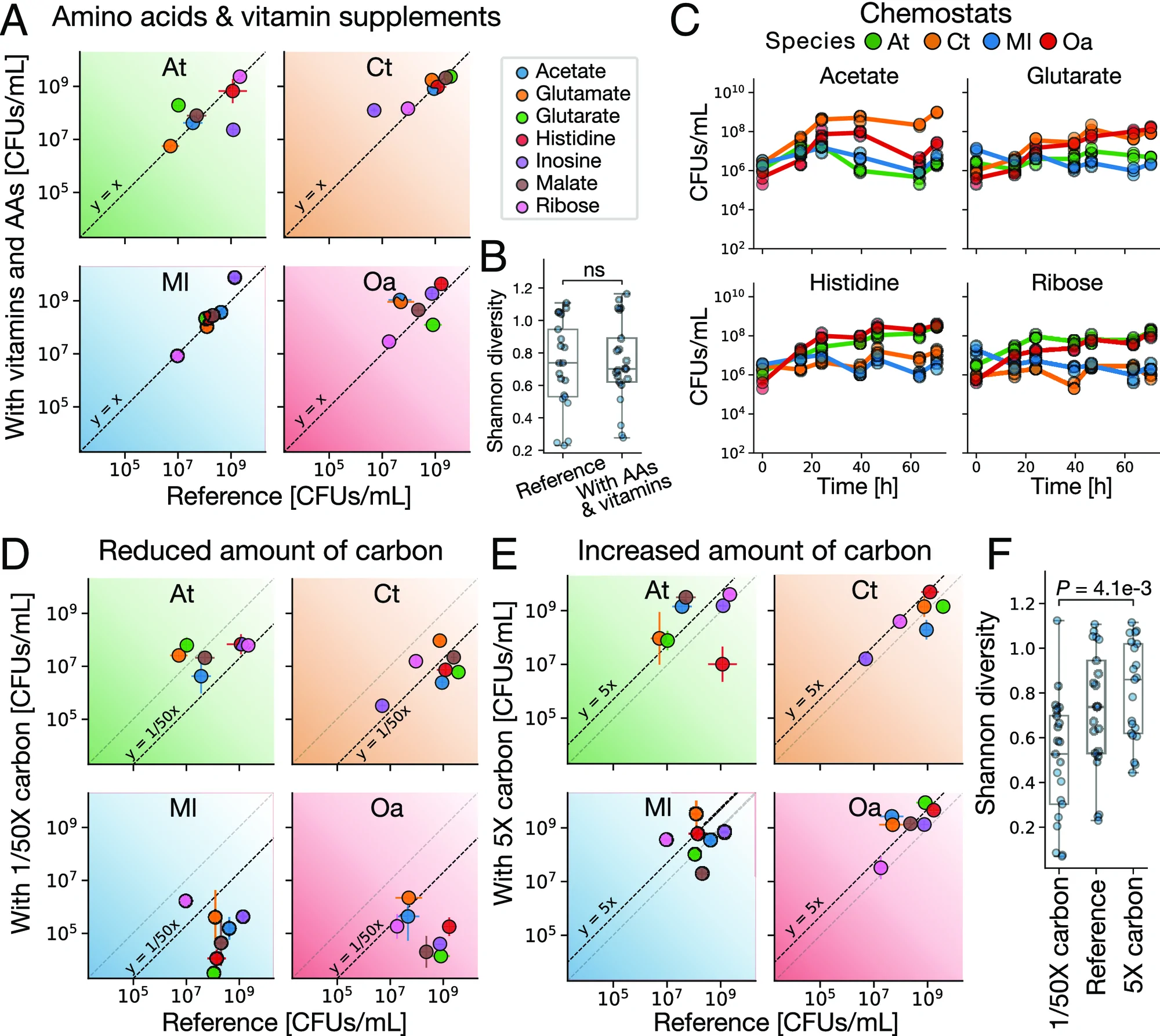
Coexistence is robust to different environments. (*A*) Effect of adding vitamins (thiamine and biotin) and amino acids (proline and cysteine) to seven reference single carbon source conditions, here shown as final population sizes with (y-axis) or without (x-axis) supplements after eight transfers (mean ± SD, n=3). (*B*) We find no significant difference in Shannon diversity between the reference conditions and the environments with additional vitamins and amino acids (*P*= 0.72, Mann–Whitney U). (*C*) Continuous cultivation in chemostats did not disrupt species coexistence, despite considerably lower abundances (*SI Appendix*, Fig. S4*D*). (*D*) All four species also coexisted with a 50-fold reduction in carbon, but these conditions seemed to disfavor the auxotrophs (Ml and Oa) with abundances in most cases well below the naive expectation of 50X less than in the reference conditions. (*E*) Ct went immediately extinct in the 5X malate condition. Otherwise all four species coexisted, but with significant shifts in abundances compared to the reference condition. (*F*) We found a significant difference in Shannon diversity between the 1/50X and 5X carbon conditions (Kruskal–Wallis H-test followed by Dunn’s post hoc test, Benjamini–Hochberg correction).

To investigate the mechanisms driving this robust coexistence, we systematically tested several ecological hypotheses that might explain how richness is maintained beyond expectations from the external resource supply. Given that auxotrophic dependencies appeared to shape community structure, we first hypothesized that these requirements were the primary stabilizing force. We therefore repeated the community assembly experiment with seven of the same single carbon source environments, but now also supplemented with the amino acids (cysteine and proline) and vitamins (thiamine and biotin) needed by Oa and Ml. Relieving these metabolic dependencies was not sufficient to break coexistence nor change the average diversity (Fig. 2 *A* and *B* and *SI Appendix*, Fig. S4*A*), but we found a reduced, and no longer significant, increase in Shannon diversity in the environments with an abundant auxotroph (>25%, *P*= 0.11, Mann–Whitney U, *SI Appendix*, Fig. S4*B*). Note that both proline and cysteine can also be used as carbon sources, and all four species also coexisted when only provided the amino acid and vitamins supplements (*SI Appendix*, Fig. S4*C*). As expected by their metabolic requirements, Oa reached higher steady-state densities in supplemented conditions, and Ml showed a similar expansion in inosine, the only carbon source on which it exhibits the highest effective growth rate. Despite these shifts in individual species performance, the persistence of all four species across all supplemented environments suggests that the underlying stabilizing mechanisms extend beyond simple vitamin or amino acid dependencies.

We next addressed whether the periodic resource fluctuation in the consecutive batch cultures could explain the observed coexistence. Theoretical studies have shown that resulting temporal fitness advantages can support increased species coexistence (39–42), for example due to tradeoffs between growth rate and lag-time (43) or growth rate and resource affinity (44). To test this, we repeated the community assembly experiment in four single carbon source environments in chemostats with continuous inflow of nutrients and outflow of the microbial culture. Species abundances increased in the chemostats to reach steady-states within 39 h, with all species coexisting in all four conditions (Fig. 2*C*). The steady-state abundances in chemostats were correlated with the steady-state abundances in the batch transfer community assembly (Pearson ρ= 0.67, P= 4.9e-3), but abundance ranks shifted compared to the batch cultures and abundances were consistently lower in the chemostat experiment (*SI Appendix*, Fig. S4*D*), reflecting the transition from favoring species with high maximum growth rate and short lag-time in batch cultures to favoring the species able to sustain the imposed dilution rate at the lowest resource concentration in the chemostats (14).

Finally, we explored how interaction strength influenced coexistence. Previous research has demonstrated the importance of interaction strength for the stability and diversity of microbial communities, and shown that interaction strengths can be modulated by total resource availability (45, 46). We therefore repeated the community assembly experiment with a fivefold increase (5X) or a 50-fold reduction (1/50X) in carbon concentration. Except in the 5X malate condition, where Ct went extinct immediately after the first transfer, all species coexisted in both the high and low resource concentrations (Fig. 2 *D* and *E* and *SI Appendix*, Fig. S4*A*). Community compositions were, however, strongly affected by the change in resource concentration, potentially reflecting rate vs. resource affinity differences, toxic effects in the 5X conditions (46), or differences in metabolic byproducts and cross-feeding patterns. Given that Ct had the highest effective growth rate on malate (Fig. 1*B*), its rapid extinction in the 5X malate condition was unexpected. To explore this result, we monitored Ct monocultures in 5X malate: Ct grew to high abundances over the first 24 h, but then the population collapsed over the next 48 h. At this time-point, the pH exceeded 10 despite the medium being buffered (compared to ∼8 in 1X malate), explaining the sudden death. After a 1/100 transfer and a second 72-h batch culture, the population of Ct was below the detection limit, mirroring results from the community assembly experiment (*SI Appendix*, Fig. S5), and revealing another example of ecological suicide (47).

We observed significantly lower diversity in the 1/50X environments compared to the 5X environments (Fig. 2*F*), contrasting with previous results showing increased diversity in low nutrient environments (45, 46). Interestingly, the low concentrations seemed to disfavor the auxotrophs, with most abundances below the naive expectation of 1/50 of the abundance in the reference condition, potentially suggesting that cross-feeding is weaker when resources are scarce. While a general correlation between growth rate and metabolite release remains undescribed, overflow metabolism causing the release of fermentation end-products such as acetate indeed only occurs when resources are abundant and the growth rate is high (48). Finally, we also found that the four species coexisted in rich media (LB and TSB), with high diversity in TSB and very low diversity in LB, with Ml almost going extinct and reaching abundances comparable to our negative control without any provided carbon source (*SI Appendix*, Fig. S4*C*). Collectively, these results demonstrate that the observed four-species coexistence is remarkably robust and resilient to changes in nutrient complexity, temporal dynamics, and resource concentration.

## Cross-Feeding Explains Coexistence.

**Fig. 3.**
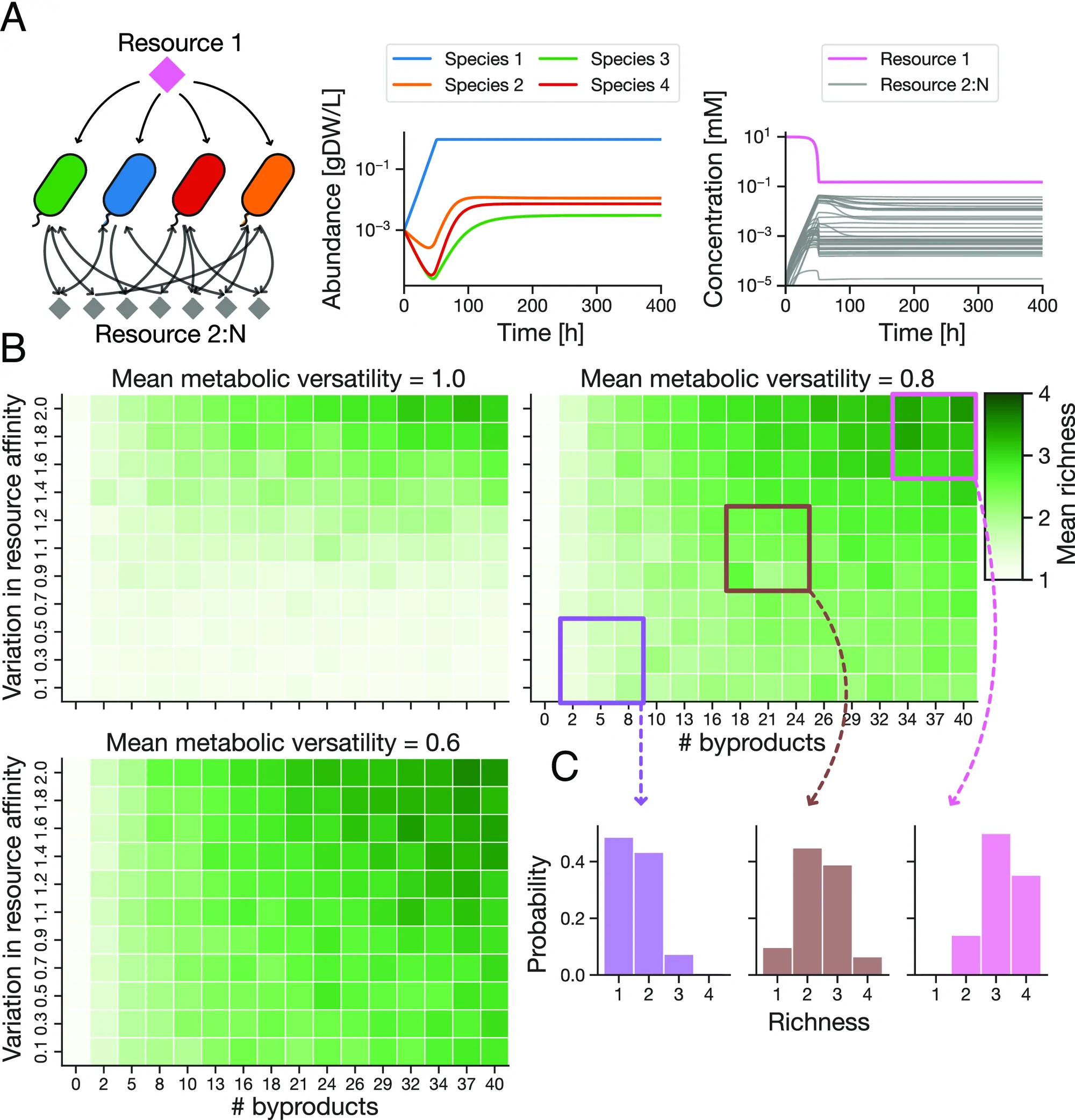
A consumer–resource model explains coexistence on a single resource. (*A*) Illustration of the consumer–resource model and an example simulation showing the simulated species and resource dynamics leading to coexistence. (*B*) The heatmaps display mean richness across 50 simulations for each combination of parameters. The variation in resource affinity is the SD of the lognormal distribution used to draw half-saturation constants. The mean metabolic versatility is the probability of each species being able to utilize each resource. (*C*) The distribution of richness across parameter regions indicated in panel (*B*).

Having excluded alternative explanations for the observed coexistence, cross-feeding emerged as the primary stabilizing mechanism. Microbial metabolite release is common and chemically diverse in in vitro cultures (20, 22, 49), and has been linked to coexistence both on the strain and species level in similar experiments (16, 18, 50). To calibrate our expectations for how cross-feeding shapes coexistence, we employed a consumer–resource modeling (CRM) framework, which explicitly describes the coupled dynamics of species and resources (51, 52). Building on previous work demonstrating that cross-feeding can promote coexistence and that CRMs capture relevant patterns of microbial communities (18, 53), we used this framework to quantify the influence of specific microbial traits on community richness. Our CRM simulates growth, resource depletion, and byproduct accumulation as a function of species’ resource affinities, maximum uptake rates, and metabolite release profiles and thus captures both resource competition and cross-feeding (Fig. 3). While we measured growth rates across the four species (*SI Appendix*, Fig. S2*C*) to parameterize our CRM with realistic uptake rates (*Materials and Methods*), the simulations capture generic species and resource pools, rather than a precise mapping of the species and resources used in our in vitro experiments. We began by screening random four-species communities on a single provided resource across three levels of metabolic versatility, defined as the average fraction of resources a species can utilize. High or low metabolic versatility can be intuitively understood as communities composed predominantly of generalists or specialists, respectively. Within each level of versatility, we systematically varied both the number of metabolic byproducts and the variability in resource affinities.

Our simulations revealed that community richness depends strongly on the interplay between metabolic versatility and variation in resource affinities (Fig. 3*B*). Intuitively, reduced versatility combined with high affinity variation increases the probability of each species specializing on a “private” metabolic niche, thereby promoting coexistence. Even at a moderate number of byproducts and moderate resource affinity variation [below the SD of measured, log-transformed resource affinities of 1.6 (54)], the model frequently produced coexisting four-species assemblages on a single primary resource. When the number of byproducts and variation in resource affinities were large, and metabolic versatility moderate, coexisting four-species communities were common (Fig. 3*C*).

**Fig. 4.**
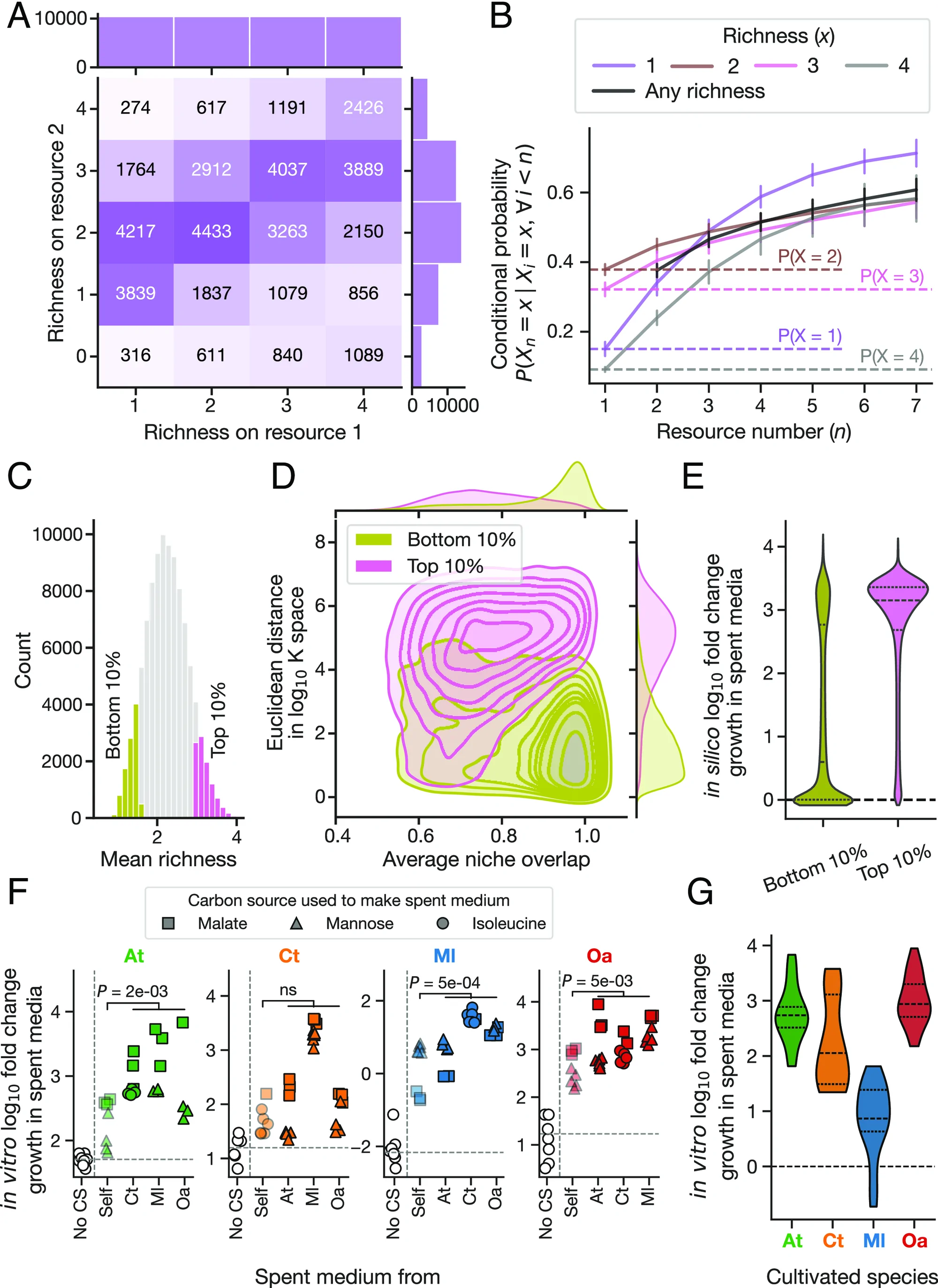
Consumer–resource models reveal hallmarks of robust community richness. (*A*) Richness of CRM communities is highly correlated between provided resources, as displayed here for resource 1 and resource 2 (Kendall’s τ=0.25, *P* < 1e-308, N=41,640). This dataset was subsampled to a uniform distribution of richness outcomes from 1 to 4 on resource 1 to simplify interpretations. (*B*) Conditional probability of maintaining a specific richness on resource n, given consistent richness across all preceding resources. The black line represents the chance of having the same richness on resource *n* as on all preceding resources. The dashed lines represent the unconditional probability of each richness. (*C*) Distribution of mean richness across seven provided resources for CRM communities, highlighting the most rich (top 10%) and least rich (bottom 10%) used to further understand the hallmarks of consistently rich communities. (*D*) The least and most rich CRM communities are well separated on the Euclidean distance in log_10_ K space and average niche overlap, K denoting the vector of resource affinities (*Materials and Methods*). (*E*) In contrast to the least rich communities, the top 10% most rich communities consistently achieve high growth yields when cultivated on simulated spent media. (*F*) In vitro, we also found that the species in general grew well in spent medium from one of the other species, and even in its own spent medium, here shown as log_10_ fold change in cell population [CFUs/mL] from inoculation to end of batch culture (after 72 h). Statistical tests of growth in own spent media vs. spent media from partners using Mann–Whitney U. (*G*) Violin plots summarizing the distribution of in vitro growth in spent media for each of the four species [summarizing data in panel (*F*)], qualitatively recapitulating CRM simulations in spent media for the most rich communities shown in panel (*E*).

To better understand the properties determining a community’s ability to maintain high richness across environments, we conducted a new set of simulations across a parameter subset expected to yield a diverse range of richness outcomes. Rather than drawing new species pools (with new species properties) for each simulation, we iteratively simulated growth across seven different resources for each community (by changing the index of the single, provided resource in our CRM), and asked whether the richness of some communities was robust to the identity of the provided resource. Indeed, we found a significant correlation between the richness of a community grown on two different resources (Fig. 4*A*). To extend beyond pairwise comparisons, we quantified the conditional probability of a community maintaining its richness across *n* sequential resources. Consistent with the pairwise results, the conditional probability increased substantially with the number of preceding resources yielding the same richness (Fig. 4*B*). Essentially, communities that exhibited high richness in one environment tended to exhibit high richness across other environments, demonstrating that richness is largely an intrinsic property of the community itself, emerging from the network of metabolic interactions rather than the identity of the external resource supply. In contrast, species’ abundances, or abundance ranks, were only weakly correlated between two supplied resources (*SI Appendix*, Fig. S6 *A* and *B*). Rather, species’ abundance ranks were largely determined by their fitness on the provided resource (*SI Appendix*, Fig. S6*C*), which was a good predictor of the most abundant species, largely reflecting our experimental findings (Fig. 1*D*).

To identify the metabolic properties of consistently rich communities, we calculated the average richness across the seven resources and compared the top 10% with the bottom 10% (Fig. 4*C*). In the parameter space, these two groups separated by (i) Euclidean distance between log-scaled resource affinities and (ii) by niche overlap (Fig. 4*D*). Given the technical challenges of quantifying resource affinities for cross-fed metabolites in vitro, we utilized our consumer–resource framework to identify more accessible signatures of robust richness. We found that species in the most rich (top 10%) communities were consistently able to grow well on the simulated spent medium of their partners, in contrast to the least rich (bottom 10%) communities (Fig. 4*E*). We then conducted similar experiments in vitro: We cultivated each of the four species in monoculture to stationary phase on two different carbon sources, malate or mannose (for Ct we used isoleucine instead), to create spent media. These eight spent media were then used to cultivate the four species in monoculture, with growth compared against no-carbon controls. In agreement with the most diverse (top 10%) simulated communities, all four species exhibited robust growth on the metabolic byproducts of their partners (Fig. 4*F*). While self-facilitation was observed, as reported previously (18, 34, 55), growth was more pronounced when species were cultivated on the spent media of other community members (Fig. 4*F*). Across all conditions, distributions of fold-change growth qualitatively mirrored the results from the top 10% simulated communities (Fig. 4 *E* and *G*), providing empirical evidence that a network of cross-feeding interactions underpins the observed coexistence.

## Interaction Patterns Support Extensive Cross-Feeding and Metabolic Niche Separation.

**Fig. 5.**
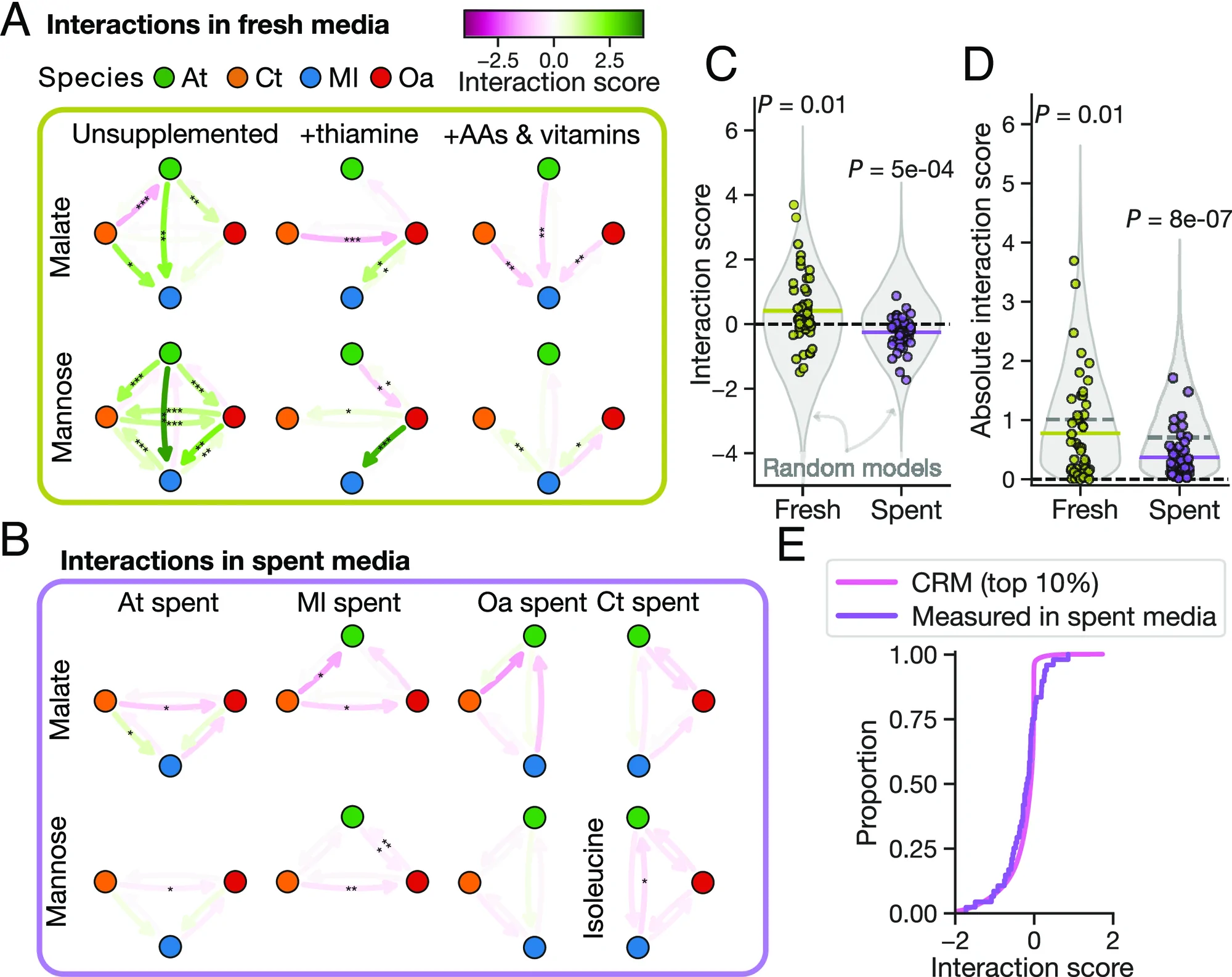
Species interactions in fresh and spent medium. (*A*) Pairwise interaction scores in fresh malate and mannose (isoleucine) media, illustrating the shift in interaction strength and sign following vitamin and amino acid supplementation. (*B*) Interaction scores in spent media with either malate, mannose, or isoleucine as the original carbon source. Interactions were quantified as the log_10_ fold-change in population size (CFUs/mL) of cocultures relative to monocultures. Statistical significance in A and B was determined on log_10_-transformed population sizes using Welch’s *t* test with Benjamini–Hochberg correction (*P < 0.05, **P < 0.01, and ***P < 0.001). (*C*) Interaction scores across fresh and spent media compared against null distributions from random models (*Materials and Methods*). Colored horizontal bars indicate mean values. A two-sided one-sample Welch’s *t* test was used to assess the significance of the mean deviation from zero. (*D*) Magnitude of interaction scores compared against null distributions from random models [as in (*C*)]. Colored and gray horizontal bars represent the median absolute scores of the experimental data and the null models, respectively. A two-sided one-sample Wilcoxon signed-rank test was used to assess whether the observed median magnitude significantly deviated from the null expectation. (*E*) Cumulative distribution functions comparing in vitro and in silico (top 10%) interaction scores in spent media.

The sign and magnitude of pairwise species interactions are key determinants of the emergent properties of microbial communities (56–58). To elucidate the association between interactions and coexistence, we quantified pairwise interaction scores by calculating the log-response ratio, defined as the $\log_{10}$ ratio of a species’ final abundance in coculture relative to its abundance in monoculture (58). Under this framework, positive interaction scores indicate facilitation, while negative scores represent competition. In fresh, unsupplemented media, our community displayed a striking dominance of positive interactions (Fig. 5*A* and Dataset S1). Not surprisingly, in mannose (where only At can grow independently, Ct cannot utilize mannose and Oa and Ml are auxotrophs), all interactions were positive with the exception of those where At served as the focal species, as it provided the primary metabolic support for the community without receiving equivalent benefits in return. Supplementing the media with amino acids and/or vitamins weakened and shifted the interactions toward negative (Fig. 5*A*), mirroring previous findings showing reduced facilitation and increased competition in more benign environments (27, 59, 60).

While interactions in fresh media elucidate dependencies and competition for the initial resources, they do not resolve how species partition or compete over metabolic byproducts in the assembled communities. We reasoned that if the species effectively partition the available byproducts, they should exhibit weak interactions in spent media. Furthermore, if the vitamins and amino acids required by the auxotrophs were already abundant in the spent media, we expected fewer strong positive interactions. Indeed, we observed a significant shift toward negative interactions in spent media (Fig. 5 *B* and *C* and Dataset S1). Interaction scores are fundamentally constrained by the yield of the growth environment, complicating the comparison of interaction strengths. Therefore, to estimate distributions of interaction scores expected by chance, we calculated interaction scores from 10,000 random mono- and coculture population sizes from lognormal distributions fitted to the range of observed values in fresh and spent media, respectively (*Materials and Methods*). In comparison to these null distributions, absolute interaction strengths were lower than expected in both fresh and spent media, but the difference was pronounced in spent media (ratio of observed to expected median: 0.78 in fresh vs. 0.54 in spent, Fig. 5*D*). Furthermore, the distribution of interactions in spent media aligned well with the distribution of simulated interactions in the top 10% rich communities (Fig. 5*E*). The consistent convergence of our experimental results with predictions from the most rich (top 10%) CRM communities suggests that our four-species community is an example of a community where metabolic interactions ensure robust coexistence across divergent environments.

## Discussion

Our results demonstrate that a synthetic four-species bacterial community can maintain robust coexistence across a vast array of nutrient environments, regardless of external resource complexity. We rule out several alternative explanations and show that this robust coexistence can be explained by niche partitioning of metabolic byproducts from the cocultivated species. It is already known that microbes create chemically rich exometabolomes (19–22, 49), and that niche partitioning of such exometabolomes occurs in the lab (17, 18, 23) as well as in natural microbiomes (61). Here we show that such niche partitioning is sufficient to support coexistence irrespective of the external resource supply and hypothesize that such interaction-driven diversity is the norm rather than an exceptional feature of our specific four-species community. As such, our findings mark a conceptual shift from viewing microbial diversity as a function of the external environment to viewing it as a property driven by the traits of the community members. It is important to note, however, that the extrapolation of these findings to natural communities requires careful consideration. As natural ecosystems typically contain hundreds or thousands of different species (62, 63), it remains unknown whether the diversity of metabolic byproducts is sufficient to provide a stabilizing niche for every member of such highly complex assemblages.

A central finding of this study is that robust coexistence appears to be an intrinsic feature of the community itself—ingrained in the species’ metabolic byproducts and the differences in their catabolic capacities. While metabolic versatility is a classic determinant of coexistence (64, 65), our simulations suggest that variance in resource affinities plays a key role in maintaining richness across environments. In accordance with these simulations, experimental data show substantial variation in resource affinities within and between species (54) and demonstrate that such variation drives niche partitioning of provided resources (66). High-throughput quantification of resource affinities across diverse nutrients and species could further illuminate how natural variability in these traits enables coexistence. Such efforts may eventually allow for in vitro experiments with communities that span a broad range of affinity profiles. Our experiments indicate that our specific four-species community exhibits the hallmarks of consistently rich communities as predicted from CRMs. While some of these hallmarks, such as affinities for cross-fed metabolites, are cumbersome to measure and difficult to scale to the design of more complex synthetic communities, spent-medium assays are straightforward and potentially equally informative. As the design of synthetic communities becomes increasingly relevant for human health, agricultural, or industrial applications, these findings suggest a feasible avenue toward designing communities whose coexistence remains robust to the variation found in such environments.

## Materials and Methods

### Strains, Media, and Culturing Conditions.

All experiments were conducted with one or more of these four strains previously isolated from and cultivated in metal-working fluids: *Agrobacterium tumefaciens* MWF001 (At), *Comamonas testosteroni* MWF001 (Ct), *Microbacterium liquefaciens* MWF001 (Ml; now classified as *M. maritypicum*), and *Ochrobactrum anthropi* MWF001 (Oa; now classified as *Brucella anthropi*) (27, 32). These species can be distinguished on selective media and quantified by plating and counting of colony-forming units (CFUs) (33).

Most experiments were conducted in M9 minimal medium with different carbon sources. Unless otherwise stated, the amount of carbon in each medium was normalized to 90 mM of carbon atoms. The different M9 minimal media were prepared by mixing 10 mL of autoclaved 10X M9 salts (Sigma-Aldrich, US, M6030-1KG), 1 mL of 50X HMB (35), 5 mL of 10X carbon source stock and 34 mL of MilliQ water. Finally, all media were pH-adjusted to pH 7.4 (with NaOH/HCl) and sterile-filtered.

For all experiments, strains were streaked from glycerol stocks onto Tryptic Soy Agar (TSA) and incubated at 28 ^°^C for 2 d. Single colonies were used to inoculate 10 mL Tryptic Soy Broth (TSB) in 50 mL Erlenmeyer flasks and grown overnight. These precultures were then diluted to an OD_600_ of 0.05 in 20 mL fresh TSB in 100 mL flasks and incubated for 3 h (28 ^°^C, 200 rpm). Prior to inoculation of experimental assays, 10 mL aliquots of these cultures were washed by centrifugation (3,220 rcf, 6 min), the supernatant removed, and the pellet resuspended in 1 mL carbon-free M9 minimal medium. The resuspended cells were centrifuged again (8,000 rcf, 4 min), the supernatant discarded, and the pellet resuspended in 1 mL carbon-free medium. This last wash step was repeated once.

### Growth Phenotyping.

We characterized the metabolic repertoire and dependencies of the four species through cultivations in M9 minimal media. Previous laboratory studies identified Oa as a thiamine auxotroph on solid media. Additionally, the inability to cultivate Ml in monoculture suggested that this species was also an auxotroph.

We first verified that Ml was cultivable in 15 mM glucose M9 medium when supplemented with a vitamin mix composed of biotin (Acros organics, D(+)-Biotin, 96%), thiamine hydrochloride (Fluka, Vitamin B1 hydrochloride #95160/25g; herein referred to as thiamine) and niacin (Sigma-Aldrich, Nicotinic acid #72309/100g) and an amino acid mix composed of all 20 proteinogenic amino acids (*SI Appendix*, Table S1). Vitamins were added to final concentrations of 0.1 mg/L biotin, 0.5 mg/L thiamine, and 1 mg/L niacin. All amino acids were supplemented to a final concentration of 0.15 g/L each. We initially used GapMind (67) to identify potential amino acid auxotrophies in Ml. GapMind flagged histidine, lysine, tyrosine, and tryptophan as potential auxotrophies for Ml, but supplementing the glucose M9 medium with the vitamin mix and these four amino acids did support growth of Ml. We therefore conducted an amino acid dropout screen in glucose M9 plus vitamin mix medium featuring all 20 N-1 proteinogenic amino acid combinations in addition to all amino acids and no amino acids controls. Following the previously described preculturing protocol, Ml was inoculated at OD_600_ = 0.01 in 4 mL culture medium in straight glass tubes (15.8 cm tall, 1.5 cm diameter) for 114 h (28 ^°^C, 200 rpm, two replicates). Growth was monitored by measuring OD_600_ directly in the glass tubes at nine time points spread over the course of the experiment (NANOCOLOR VIS II, Macherey-Nagel, Düren, Germany). The N-1 amino acid dropout experiment revealed proline and cysteine as potential auxotrophies (*SI Appendix*, Fig. S1*D*). To verify these auxotrophies, we conducted a follow-up experiment with supplementation of proline, cysteine, or both (*SI Appendix*, Fig. S1*E*, three replicates, 188 h, otherwise similar experimental procedure, equipment, and parameters). In the same experiment, we also verified the vitamin auxotrophies of Ml and Oa that were predicted from the presence/absence of key genes (according to ref. 68) in the annotated proteomes obtained from NCBI under the accession code PRJNA991498 (28) (*SI Appendix*, Fig. S1*A*). In agreement with the absent genes, the growth of Ml was reduced when leaving out either biotin or thiamine, and the growth of Oa increased substantially on thiamine supplementation.

To select a set of carbon sources for further growth phenotyping, we used a computational approach to identify carbon sources that were metabolically diverse. For this purpose, we used the universal genome-scale metabolic model (GEM) for bacteria from CarveMe (69). Using this GEM, we predicted intracellular flux distributions with 509 different metabolites using parsimonious Flux Balance Analysis with growth as the objective (70). This optimization was conducted using COBRApy (71) and Gurobi (v10.0.3), setting bounds on exchange reactions to mimic aerobic growth in a minimal M9-like medium (*SI Appendix*, Table S2). The uptake rate of the carbon source was set to 10 mmol gDW^−1^ h^−1^. To identify groups of metabolites with different flux patterns we first converted fluxes to binary values (0, −1, or 1), discarded fluxes with zero variability, conducted a Principal Component Analysis (PCA) on the remaining binary flux matrix, and finally used HDBSCAN (72) to group metabolites based on principal components 1 and 2 (*SI Appendix*, Fig. S2*A*). We then selected 33 carbon sources that differed in metabolite class (amino acids, organic acids, simple sugars, alcohols, nucleoside, and others) and covered all 9 clusters (*SI Appendix*, Table S3). We then tested the ability of the 4 species to grow on each of these carbon sources in minimal M9 medium, using concentrations corresponding to 90 mM carbon atoms (except for Ct in citrate, where we used 5 mM rather than 15 mM citrate because of Ct’s long lag-time in high citrate concentrations). Additionally, for Oa we supplemented the media with 0.5 mg/L thiamine, and for Ml we supplemented the media with 0.1 mg/L biotin, 0.5 mg/L thiamine, 0.18 g/L cysteine, and 0.1 g/L proline. We also included a no-carbon-source control condition (which also included the vitamin and amino acid supplements). For these experiments, each strain was precultured and washed as described above and then inoculated to OD_600_ = 0.05 in 200 µL of media in 96-well plates and cultivated with continuous shaking (double orbital, 425 cpm) in a BioTek Synergy H1 (Agilent Technologies, Winooski, VT, USA) plate reader at 28 ^°^C for 72 h (*SI Appendix*, Fig. S2*B*).

We quantified the ability of each species to grow on each of the 33 carbon sources qualitatively (growth or no growth) and quantitatively (maximum growth rate, lag-time, yield). A carbon source was classified as growth-supporting if cultures exceeded a minimum yield (OD_max_− OD_start_> 0.05) and if this yield was statistically greater than the no-carbon control (*P* < 0.05, Welch’s one-sided *t* test, N=3). Growth parameters were extracted from the growth curves using curveball (73), but only fitting growth models until the time of maximum OD_600_. Regardless of which model curveball selects as the best fit, the common parameters are maximal growth rate ($\mu_{\max})$, the lag time ($t_{\mathrm{lag}}$), the final yield (Y), and the initial starting OD ($y_{0}$). From these four parameters, we define the effective growth rate ($\mu_{\mathrm{eff}}$) as the constant exponential rate that would be required to reach the observed yield Y from the initial density $y_{0}$ in the total time. In the absence of lag, the effective growth rate and the maximal growth rates are equal (at high resource affinity levels). With nonzero lag time, the effective growth rate is given by

$$\begin{array}{c} \mu_{\mathrm{eff}}=\mu_{\max}\left(\frac{\log\left(Y\right)-\log\left(y_{0}\right)}{\log\left(\frac{Y}{y_{0}}\right)+\mu_{\max}t_{\mathrm{lag}}}\right) \end{array}$$

This measure thus captures the joint effect of lag time and maximal growth rate on competitive ability, rather than relying on $\mu_{\max}$ alone. This is important since the relationship between these parameters has been shown to impact competitive ability (43).

### Community Assembly Experiments.

The community assembly experiment was conducted in 32 different M9 minimal media, with 16 single carbon sources, 15 pairs of carbon sources and a no-carbon control environment (Fig. 1). The four species were precultured and washed as described above. Washed cultures were combined at equal densities (each species to OD_600_= 2), vortexed, and used to inoculate all wells of a 96-well plate to an initial OD_600_= 0.03 (in 200 µL) of each species to start the first growth cycle. Each transfer of the community assembly was cultivated for 72 h in a plate reader (Biotek, Synergy H1) at 28 ^°^C with continuous shaking (double orbital, 425 cpm). On each transfer a 1/100 dilution (2 µL) was performed into a freshly made plate. However, wells with citrate or inosine as the carbon source were only diluted 1/10 because of low yield (first transfer of citrate wells was 1/100). All wells were carefully mixed using a 200 µL multiwell pipette set to 70 µL before each transfer. The abundance of each species at the end of each growth cycle was quantified by counting CFUs on selective media (33). Quantified population sizes (as CFUs/mL) were log_10_-transformed prior to statistical analyses and calculation of abundance ranks. Log_10_-transformed CFUs from the two last transfers were used to compute final abundance ranks (by comparing means) and for statistical analyses. To compare the final abundance of each species in each condition with the final abundance in the no-carbon control we used the two-sided Welch’s *t* test for At, Ct, and Oa, and the one-sample one-sided *t* test for Ml with respect to the limit of detection. The limit of detection was set to one CFU in the undiluted 5 µL volume used for CFU quantification, i.e. 200 CFUs/mL. For the second community assembly experiment (Fig. 2), seven single carbon source environments (acetate, glutarate, L-glutamate, L-histidine, inosine, malate, ribose) were repeated exactly as in the first community assembly experiments. We also included 21 conditions with the same carbon sources but either at different carbon source concentrations (5X: 450 mM and 1/50X: 1.8 mM carbon) or supplemented with vitamins (0.1 mg/L biotin, 0.5 mg/L thiamine) and the amino acids required by Ml for rapid growth (0.15 g/L proline, 0.15 g/L cysteine). Finally, we also included LB, TSB, and a control with only the vitamin and amino acid supplements. All conditions were conducted in triplicates. For the second community assembly experiment, the quantified population density after the sixth transfer was used in three cases as the final population size because of issues with the CFUs from transfer 7 and 8 (two wells for Oa in Inosine 1/50×, one well for Ml in LB).

A follow-up experiment was conducted to investigate the underlying cause of Ct’s extinction in the 5X malate condition: Ct was precultured and washed as described above, and inoculated at an initial density of $1.9\times 10^{7}$ CFUs/mL into 50 mL Erlenmeyer flasks containing 10 mL of either a no-carbon control, 1X malate, or 5X malate M9 minimal medium. Cultures were grown in duplicate under batch conditions (28 ^°^C, 200 rpm). After 72 h of initial incubation, a 1/100 dilution transfer was performed into identical fresh media, followed by an additional 72-h batch cultivation phase. The abundance of Ct was quantified by CFUs on TSA after 1 d, 3 d, and 6 d. The final pH of these cultures was quantified using pH indicator strips 1-14 (Macherey-Nagel, Düren, Germany).

### Community Assembly Experiment in Chemostats.

Chemostat experiments were performed using Chi.Bio reactor modules (74) coupled to an Ismatec IPC multichannel peristaltic pump. Cultures were grown in 30 mL glass vials (Fisher Scientific, 11593532) sealed with 20 mm septa secured by hole caps (Supelco, 22 mm). Four ports were punched into each septum and fitted with PTFE tubing (ID 1/16 in, OD 1/8 in; Altec 01-96-1702) to provide lines for medium inflow, effluent outflow, sampling, and aeration/pressure release. Fresh medium was supplied from a 0.5 L glass reservoir to the growth vial using 2-stop Tygon E-Lab tubing (ID 0.76 mm) and silicone pump tubing (bore 2.5 mm, wall 1 mm; Altec 01-93-1416/20). Effluent was removed via a PTFE outflow line connected to silicone pump tubing (bore 2.5 mm, wall 1 mm) placed directly in the peristaltic pump and routed to a waste bottle. The working volume was maintained at 20 mL by fixing the height of the PTFE outflow tube to act as an overflow at the 20 mL fill level. Cultures were aerated using an aquarium air pump (JBL ProAir a50) connected to the aeration port through an inline air filter and mixed continuously with a magnetic stir bar (10 mm; Fisher Scientific 11732513). After assembly (vial, tubing, and feed/waste bottles), the complete setup was sterilized by autoclaving prior to inoculation. The peristaltic pump was programmed to a flow rate of 33 µL min^−1^, corresponding to a dilution rate of d=0.1 h^−1^ for a 20 mL working volume, and reactors were maintained at 28 ^°^C.

Five minimal-medium conditions were prepared, each standardized to a total of 90 mM carbon atoms, using ribose, sodium acetate, histidine, or glutaric acid as the sole carbon source, as well as a no-carbon control. At, Ct, Ml, and Oa were grown as described above. Prior to inoculation, cultures were adjusted and combined to assemble four-species communities with equal starting abundances and a total initial density of OD_600_ = 0.03 in each medium condition. Chemostats were inoculated on day 0 with the prepared community. From day 1 to day 4 after inoculation, cultures were sampled twice daily by withdrawing 700 µL through the septum, preparing 10-fold serial dilutions, and spot-plating 5 µL drops of each dilution on selective agar for counting CFUs of each species (see above).

### Mono- and Cocultures in Fresh and Spent Media.

Spent media were created in the following way: The four species were precultured and washed as described above. Then, the species were inoculated (in monoculture) to OD_600_ = 0.05 in 10 mL of the M9 media with glutamate, mannose, glycerol, isoleucine, or malate and cultured for 72 h (50 mL Erlenmeyer flask, 200 rpm, 28 ^°^C). Additional thiamine was included in Oa media, and additional thiamine, biotin, proline, and cysteine were included in Ml media (concentrations as in the second community assembly experiment). Optical densities were measured at 70 h and 72 h to ensure that the cultures had reached stationary phase (OD_600_ readings were unchanged between these time points). The spent culture media were filtered (0.22 µm pore size) and stored in falcon tubes at −20 ^°^C until use. Sterility was verified by plating filtered media on TSA (28 ^°^C, >48 h); any contaminated batches were refiltered and retested prior to use.

Mono- and cocultures in fresh and spent media were conducted in the following way: The four species were precultured and washed as described above, and then inoculated (either in monoculture or in pairs) to OD_600_ = 0.001 (for each species) in 200 µL of spent media in 96-well plates (see Dataset S2 for an overview of all combinations). All experiments were conducted in triplicates. The experiment with cultivations in spent media based on mannose/isoleucine was repeated once because of issues with certain CFUs, and our data include results from both experiments. Inoculum sizes were also quantified as CFUs. The cultures were incubated in a plate reader (Biotek, Synergy H1) at 28 ^°^C for 72 h with continuous shaking (double orbital, 425 cpm). Final populations of the different species were quantified as CFUs by serial dilutions and plating on selective media as described above.

### Quantification of Interactions.

Interactions in fresh and spent media were quantified as the log-response ratio (58) and the significance of each interaction was assessed using the two-sided Welch’s *t* test to compare CFUs in mono- and coculture. CFUs were log_10_ transformed prior to quantification of interaction strengths and significance. Thus, the interaction ($Y_{\mathrm{ij}}$) measuring the effect of species j on focal species i was calculated for each condition and for each partner j as $Y_{\mathrm{ij}}=\bar{\log_{10}\left(X_{i}^{co_{\mathrm{ij}}}\right)}-\bar{\log_{10}\left(X_{i}^{\mathrm{mono}}\right)}$, with $\bar{\log_{10}\left(X_{i}^{co_{\mathrm{ij}}}\right)}$ and $\bar{\log_{10}\left(X_{i}^{\mathrm{mono}}\right)}$ denoting the mean of log-transformed CFUs in coculture and monoculture, respectively.

To quantify expected distributions we used random models to create null-distributions of interaction scores in two ways: 1) We fitted normal distributions to match the 5% and 95% percentiles of the log_10_-transformed measured CFUs in fresh and spent media, respectively. From each distribution, we sampled 10,000 pairs of random log_10_-transformed CFU values (representing final abundance in mono- and coculture) and calculated the corresponding distribution of interaction scores. 2) We sampled with replacement 10,000 pairs of log_10_-transformed CFU values from the pool of measured values (for fresh and spent media separately), and calculated the distribution of interaction scores from these pairs. For the statistical comparisons of the magnitude of interaction scores, both approaches gave similar results and the same conclusion. The measured and fitted distributions of log_10_-transformed CFUs and a comparison between measured interactions and null distributions from the second approach are shown in *SI Appendix*, Fig. S7 (corresponds to Fig. 5 *C* and *D*).

### Consumer–Resource Model.

The consumer–resource model presented here builds upon Robert MacArthur’s classic framework (51, 75), incorporating recent modifications that extend the model to include metabolic cross-feeding (18, 23, 53). Briefly, the framework models the abundance of both species and resources in a chemostat-like system with in-flow of resources and out-flow of cells and resources, and the growth of each species is a function of its nutrient preferences, resource concentrations, and metabolite release.

We denote the number of species as N (indexed by i=1,...,N) and the number of resources as M (indexed by j=1,...,M). Each species is characterized by a resource uptake matrix $J_{\mathrm{ij}}$ which represents the rate at which species i consumes resource j, and a metabolite release matrix $D_{\mathrm{ijk}}$ that describes the transformation of resource j to resource k for species i upon metabolite release. The parameters in $J_{\mathrm{ij}}$ are calculated from a maximum uptake rate matrix $C_{\mathrm{ij}}$ and a substrate affinity matrix $K_{\mathrm{ij}}$ according to the Monod equation (76):

$$\begin{array}{c} J_{\mathrm{ij}}=C_{\mathrm{ij}}\frac{R_{j}}{K_{\mathrm{ij}}+R_{j}}. \end{array}$$

All species in the following simulations are given the same values for metabolite leakage (l) and growth yield g. Metabolite leakage defines the fraction of consumed resources that are released back into the environment and the growth yield defines the conversion of resource amount to biomass. For all simulations, all resources were assumed to have the same energy content and we neglected cellular maintenance. The per capita growth of each species is then described by

$$\begin{array}{c} \frac{1}{X_{i}}\frac{dX_{i}}{\mathrm{dt}}=g\left(1-l\right)\sum_{j}J_{\mathrm{ij}}-d. \end{array}$$

Here, $X_{i}$ is the population density (in gDW/L) and d is the dilution rate of the system. Resource concentrations (in mM) are then described by the following equation:

$$\begin{array}{c} \frac{dR_{j}}{\mathrm{dt}}=\left(R_{j}^{0}-R_{j}\right)d-\sum_{i}X_{i}J_{\mathrm{ij}}+l\sum_{i=1}^{N}\sum_{k=1}^{M}N_{i}J_{\mathrm{ik}}D_{\mathrm{ikj}}. \end{array}$$

Here, $R_{j}^{0}$ represents the influx concentration of resource j. Parameters for the consumer–resource matrices C, D, and K were sampled from random distributions using NumPy (77) and SciPy (78). For the consumer uptake matrix C, maximum uptake rates were sampled from a lognormal distribution,

$$\begin{array}{c} C_{\mathrm{ij}}∼\text{LogNormal}\left(\mu ,\sigma \right), \end{array}$$

where μ=0.37 and σ=0.91 are the mean and SD of the log-scaled distribution, respectively. This distribution was fitted to experimentally estimated monoculture maximum uptake rates across the four species on single carbon sources (*SI Appendix*, Fig. S2*B*). Maximum uptake rates were derived from measured growth rates by assuming that the maximum growth rate ($v_{\text{max}}$) is a function of the maximum resource uptake rate ($c_{\text{max}}$), yield (g), and leakage rate (l):

$$\begin{array}{c} c_{\text{max}}=\frac{v_{\text{max}}}{g(1-l)}. \end{array}$$

For all parameter estimations and consumer–resource model (CRM) simulations, we assumed a constant leakage rate l of 0.1 and a yield g of 0.1 gDW/mmol, yielding uptake rates in units of mmol/gDW/h. To filter out simulations resulting in global community washout, we enforced a minimal threshold for the maximum uptake of the primary externally supplied carbon source (j=0) across the four species:

$$\begin{array}{c} \max_{i}\left(C_{i0}\right)\cdot g>2d. \end{array}$$

To construct the metabolic byproduct allocation matrix D, initial values were sampled from a uniform distribution ($D_{\mathrm{ijk}}∼\text{Uniform}\left(0,1\right)$). Diagonal elements were set to zero ($D_{\mathrm{ijj}}=0,\forall i\in N,\forall j\in M$) to prevent a resource from leaking into itself. Finally, rows for each species’ D matrix were normalized to satisfy thermodynamic mass conservation:

$$\begin{array}{c} \sum_{k}D_{\mathrm{ijk}}=1,\;\forall i\in 1,...,N,\;\forall j\in 1,...,M. \end{array}$$

Resource affinities $K_{\mathrm{ij}}$ were sampled from a lognormal distribution ($K_{\mathrm{ij}}∼\text{LogNormal}\left(\mu ,\sigma \right)$), with fixed mean μ=−1 (in log-space) and varying σ.

### Simulating Four-Species Microbial Communities.

Although empirical cultivation data from the four species were used to parameterize the lognormal distribution for uptake rates, these simulations were not intended to replicate the exact community growth of these specific taxa. Consequently, resources in the CRM represent a generic nutrient pool rather than metabolites with explicit chemical or metabolic properties.

We ran two large screens of CRM simulations, both with similar CRM parameters but for distinct purposes. In both CRM screens, continuous cultivations of the four-species communities were simulated for 2,000 h at a dilution rate of 0.1 h^−1^, with initial species abundances set to $10^{-3}$ gDW/L, and both the initial and influx resource concentrations set to 10 mM. Only one resource was provided in each simulation. Simulation convergence was evaluated based on the net change in species abundance during the final time step. A simulation was classified as converged if no single species abundance changed by more than $10^{-7}$ gDW during the last time step (Δt=0.1 h). Nonconverging simulations or those experiencing numerical integration failures were discarded from downstream analysis.

We simulated four-species communities with different levels of metabolic versatility and different levels of variation in resource affinities. We define metabolic versatility as the fraction of nonzero entries in $C_{\mathrm{ij}}$ for each species i. Zero entries in $C_{\mathrm{ij}}$ were selected randomly according to the given metabolic versatility ($P(C_{\mathrm{ij}}=0)=1-\text{metabolic}\;\text{versatility}$). In the first screen, we ran simulations with metabolic versatilities of 0.6, 0.8, and 1.0, resource affinities $K_{\mathrm{ij}}∼\text{LogNormal}\left(\mu ,\sigma \right)$ with μ=−1 and σ values between 0.1 and 2, and between 0 and 40 byproducts (yielding between 1 and 41 total resources; see Fig. 3 for all parameter combinations). Fifty simulations were run for each parameter combination.

In the second screen, we generated 10,000 unique CRM realizations. For each realization, parameters were randomly sampled: The number of resources was drawn from a discrete uniform distribution on the interval [15,30], metabolic versatility from a uniform distribution on [0.6,1], and resource affinity σ from a uniform distribution on [0.1,2]. For each CRM realization we iteratively simulated growth with resource $R_{j}$ as the single, externally supplied resource, with j from 1 to 7.

### Simulating Growth and Interactions in Spent Media.

To investigate how metabolic modifications to the environment enable growth and alter subsequent species interaction, we simulated spent-medium cultivations using parameter sets from the top 10% and bottom 10% of richness outcomes in our secondary CRM screen. Spent medium was created by simulating monoculture growth for each of the four species from the top 10% and bottom 10% sets of communities for seven iterations (each iteration on a different provided resource, j={1,...7}) for 200 h from an initial population of $10^{-5}$ gDW/L, yielding a total of 640,000 unique monoculture simulations. The resource concentrations at the end of each simulation were stored and used to initialize the resource pool of subsequent CRM simulations to quantify spent-medium growth of the other three species. However, we discarded simulations where more than 10% of the initial resource was still present to ensure that the simulated growth was indeed a result of byproducts. This filtering reduced the number of unique spent media to 421,090. Although we simulated batch culture dynamics, we imposed a minimal dilution rate of $10^{-3}$ to improve numerical stability and prevent the ODE solver from stalling at infinitesimally small step sizes.

Batch cocultures in spent media for pairwise combinations were simulated using similar parameters as for the monocultures in spent media. A subset of the spent media was used for this purpose (j={1,2,3}) and we only simulated pairs where the spent media originated from a third species (i.e. for spent media we simulated three pairs). Pairwise interactions within the spent media were quantified by the log-response ratio matching experimental metrics. The interaction score ($Y_{\mathrm{ij}}$), defining the directional effect of partner j on focal species i, was calculated as

$$\begin{array}{c} Y_{\mathrm{ij}}=\log_{10}\left(X_{i}^{{\text{co}}_{\mathrm{ij}}}\right)-\log_{10}\left(X_{i}^{\text{mono}}\right), \end{array}$$

where $X_{i}^{{\text{co}}_{\mathrm{ij}}}$ and $X_{i}^{\text{mono}}$ represent the final abundances of focal species i in coculture with j and monoculture, respectively.

## Supplementary Material

Appendix 01 (PDF)

Dataset S01 (XLSX)

Dataset S02 (XLSX)

## Data Availability

The strains used in this study are available on request. All data and code used for simulations and data analyses are available on GitHub (79). A permanent archive of this repository has been deposited to Zenodo (80).
