# Supplementary material for: Cross-feeding enables robust coexistence between four bacterial species
Source: Proc Natl Acad Sci U S A. 2026 Sep 17;123(38):e2607664123. doi: 10.1073/pnas.2607664123 (PMC13598246; doi:10.1073/pnas.2607664123)
Supplement: Supplementary file 1 [file pnas.2607664123.sapp.pdf]

Supporting Information for

Cross-feeding enables robust coexistence between four bacterial species

Snorre Sulheim, Miguel Teixeira, Eric Ulrich, Alisson Gillon, Samuele E. A. Testa, Prajwal Padmanabha, Daniel Machado and Sara Mitri

Correspondence: Snorre Sulheim (snorre.sulheim@unil.ch) and Sara Mitri (sara.mitri@unil.ch).

This PDF file includes:

Figs. S1 to S7
Tables S1 to S3
Legends for Dataset S1 to S2

Other supporting materials for this manuscript include the following:

Datasets S1 to S2

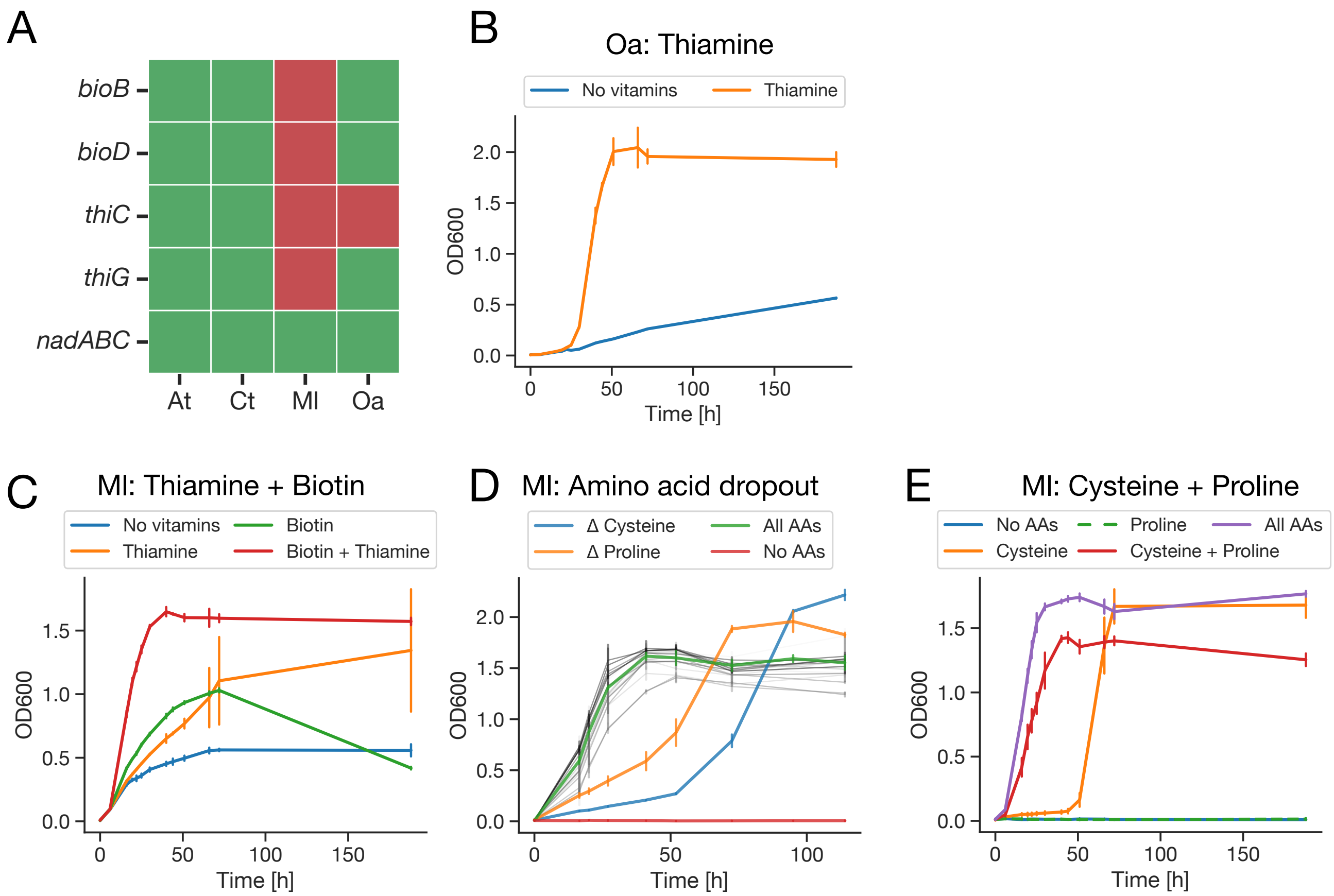

Fig. S1. Determining auxotrophies of Oa and Ml. A) Presence/absence of key genes for biotin (*bioB* and *bioD*), thiamine (*thiC* and *thiG*) and niacin (*nadA*, *nadB* and *nadC*) biosynthesis in the four species based on the annotated proteome. B & C) Evaluating vitamin auxotrophy predictions based on proteome annotations of Oa and Ml. Thiamine was needed to support rapid growth and high yield of Oa. Both biotin and thiamine were needed to support full growth rate and yield for Ml. All media here was based on a glucose M9 medium, and for the Ml cultivation all 20 proteinogenic amino acids were supplemented. D) Amino acid dropout screen to identify the amino acid auxotrophies of Ml. Ml only showed reduced growth in the ∆proline and ∆cysteine conditions. Other dropouts are shown in light gray. All media here, and in panel C, was based on a glucose M9 medium supplemented with biotin, thiamine and niacin. E) Confirmation of Ml auxotrophies identified in amino acid dropout screen. While external supply of cysteine is strictly needed for Ml's growth, the lack of proline induces a 48-hour lag phase followed by rapid growth.

Supplementary Figures

Supplementary Tables

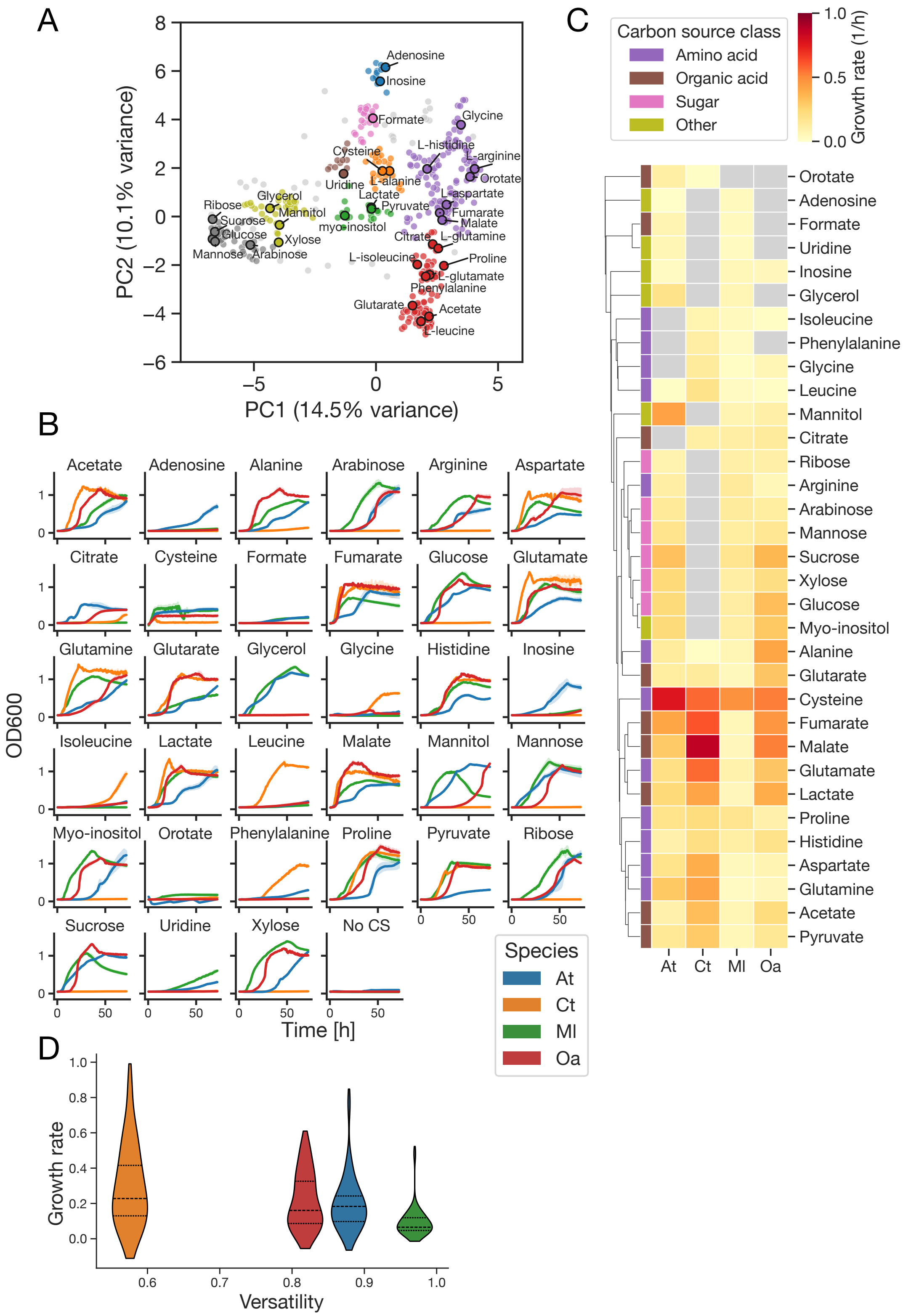

Fig. S2. Growth phenotyping on 33 carbon sources. A) A PCA used to identify metabolically diverse carbon sources based on differences in predicted flux patterns (see Methods). By selecting carbon sources from all of the nine clusters we covered metabolic variability. Annotated carbon sources are those selected to be used for growth phenotyping. B) Growth curves of each species in monoculture for each of the 33 carbon sources. Monocultures of Ml and Oa were supplemented with essential vitamins and amino acids (see Methods). Axes are identical across all panels. C) Estimated maximum growth rates across the 33 carbon sources. Gray tiles indicate conditions where a species failed to exhibit significant growth. Growth significance was defined as a mean OD_{600} increase of ≥ 0.05 over the course of the experiment and a final OD_{600} significantly greater than the no-carbon control condition ($P < 0.05$, Welch's one-sided t-test). D) Distribution of maximum growth rates for each of the four species plotted against metabolic versatility, calculated as the fraction of utilizable carbon sources.

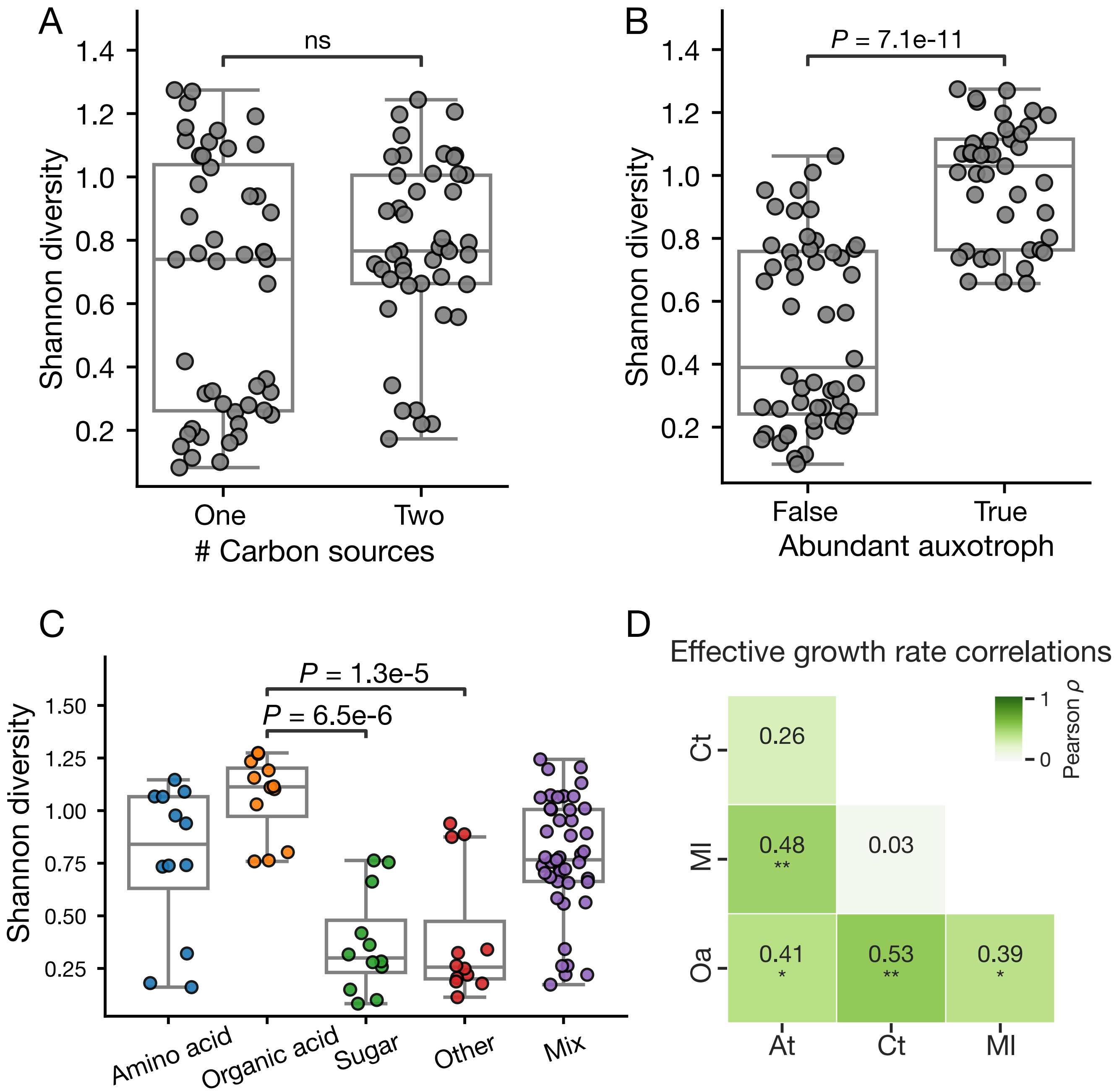

Fig. S3. Shannon diversity of community assembly experiment and growth rate correlations. A) We find no significant difference in Shannon diversity when we simply group by the number of carbon sources (P = 0.21, Mann-Whitney U). B) We find however that communities where one of the auxotrophs comprises > 25% of the total abundance to have a significantly higher Shannon diversity (P = 7.1e-11, Mann-Whitney U). C) We find also significant differences in Shannon diversity between types of carbon sources. Significance assessed using the Kruskal-Wallis test followed by Dunn's posthoc test with Benjamini-Hochberg correction. D) Pearson correlation between effective growth rates (*P < 0.05, **P < 0.01).

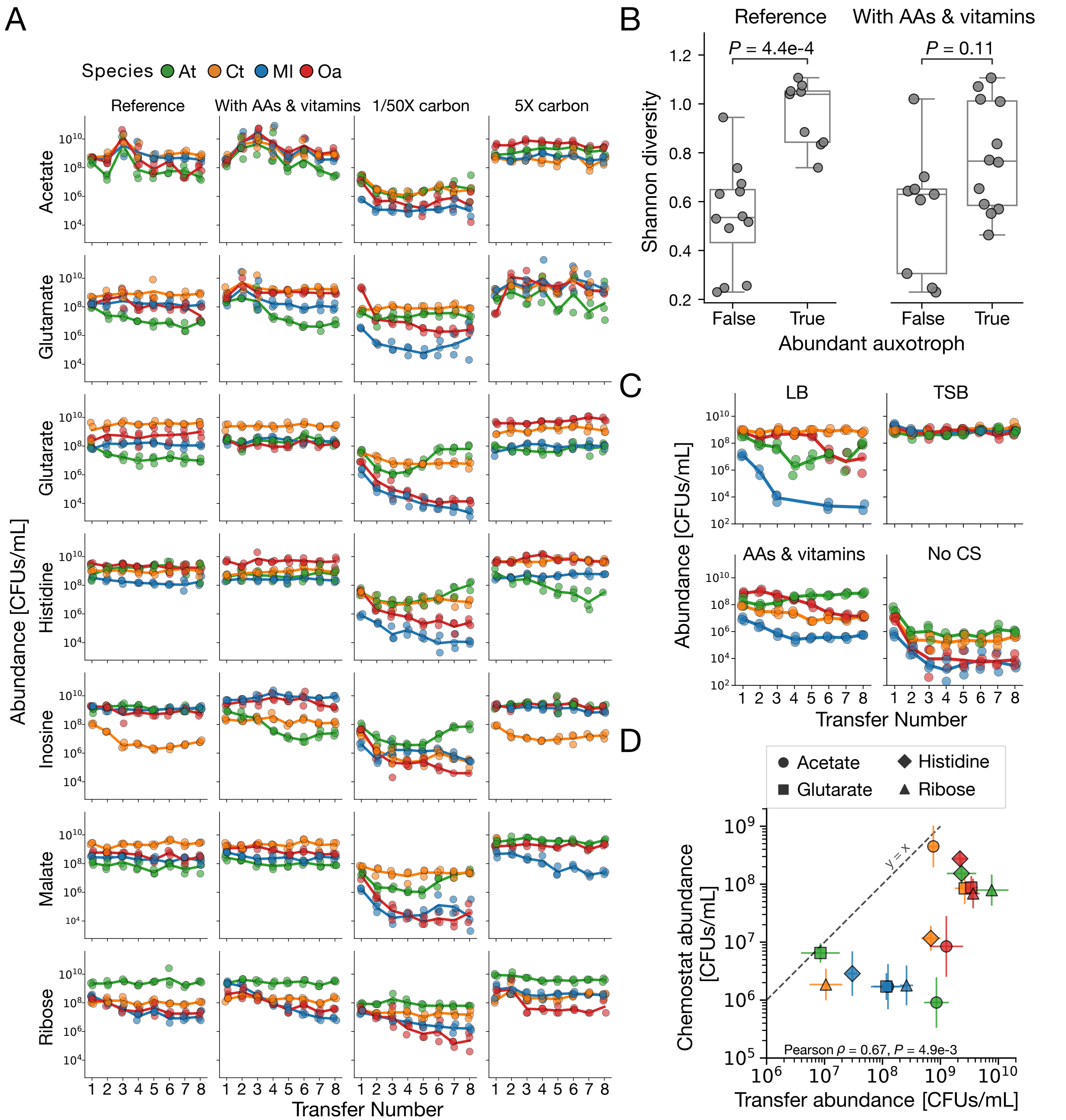

Fig. S4. Community-assembly experiments to test alternative hypotheses. A) Quantified species abundance at the end of each of the 8 consecutive batch transfers in the reference condition, with vitamin and amino acid supplementation, with reduced and increased amounts of carbon. B) A comparison of community assembly endpoint Shannon diversity with or without an abundant auxotroph (Ml or Oa >25% of the population) in the reference conditions and when supplemented with vitamins and amino acids. C) Quantified species abundance at the end of each of the 8 consecutive batch transfers in LB, TSB, in the amino acid and vitamin supplement, and in the no-carbon control. D) A comparison of final population sizes in chemostats and in the transfer experiments. The mean and standard deviation is calculated from the 2-3 samples after 41 hours for the chemostats and from the last two transfers for the transfer experiment. The Pearson correlation is calculated from the $\log_{10}$-transformed mean values.

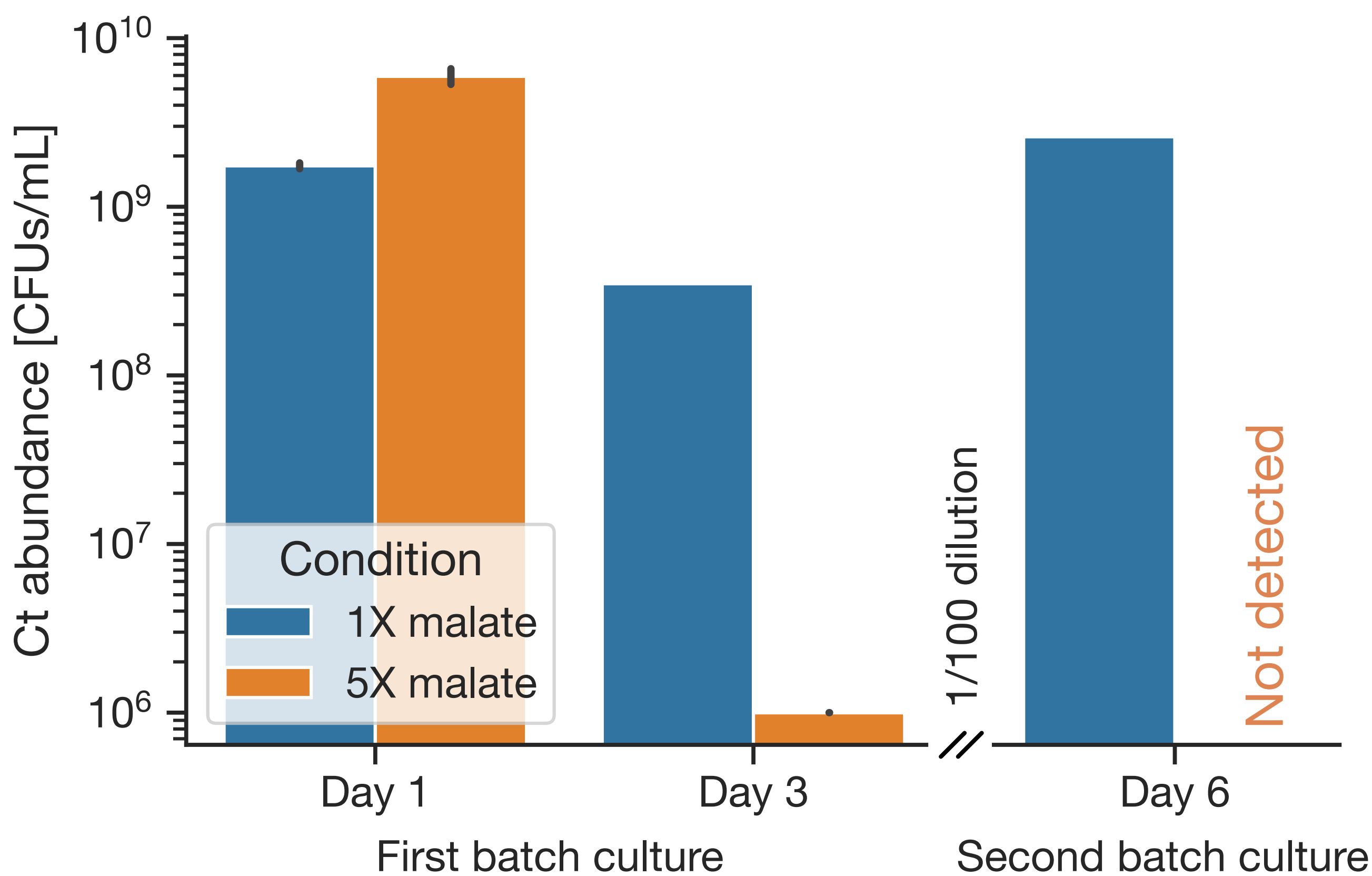

Fig. S5. Growth dynamics and population collapse of Ct in elevated resource concentrations. Ct monoculture abundances quantified over two consecutive 72-hour batch culture cycles in 1X and 5X malate media. In the 1X condition, Ct exhibits robust growth and stable survival across transfers. In contrast, the 5X condition induces initial growth to higher peak abundances followed by a rapid population collapse. This decline coincides with a severe environmental pH increase, with the final culture pH exceeding 10 in the 5X condition compared to $\sim$8 in the 1X condition. Following the second 72-hour batch cultivation cycle, Ct abundances fall entirely below the detection limit in the 5X malate environment.

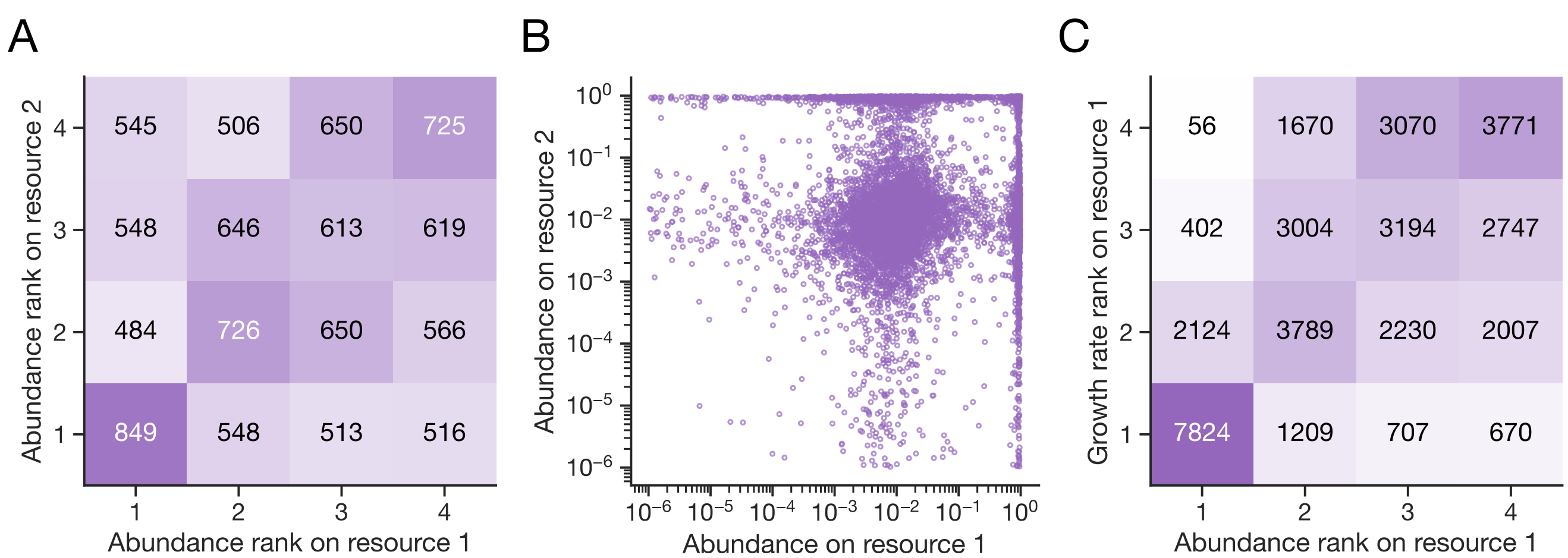

Fig. S6. Correlations in consumer-resource models of abundance ranks and abundances between resource 1 and 2, respectively, and between growth rate on the externally provided resource and abundance rank in four-species co-cultures. A) In communities where all four species coexist on both resource 1 and 2, we find a weak, positive correlations between abundance ranks (Kendall's τ = 0.09, P = 2e-26, N = 9704), and B) between $\log_{10}$-transformed abundances (Pearson ρ = 0.1, P = 9e-22, N = 9704). C) In accordance with our experimental results, we find a strong positive correlation between the growth rate rank on the externally supplied carbon source and abundance rank in four-species co-cultures (Kendall's τ = 0.50, P < 1e-308, N = 41640)

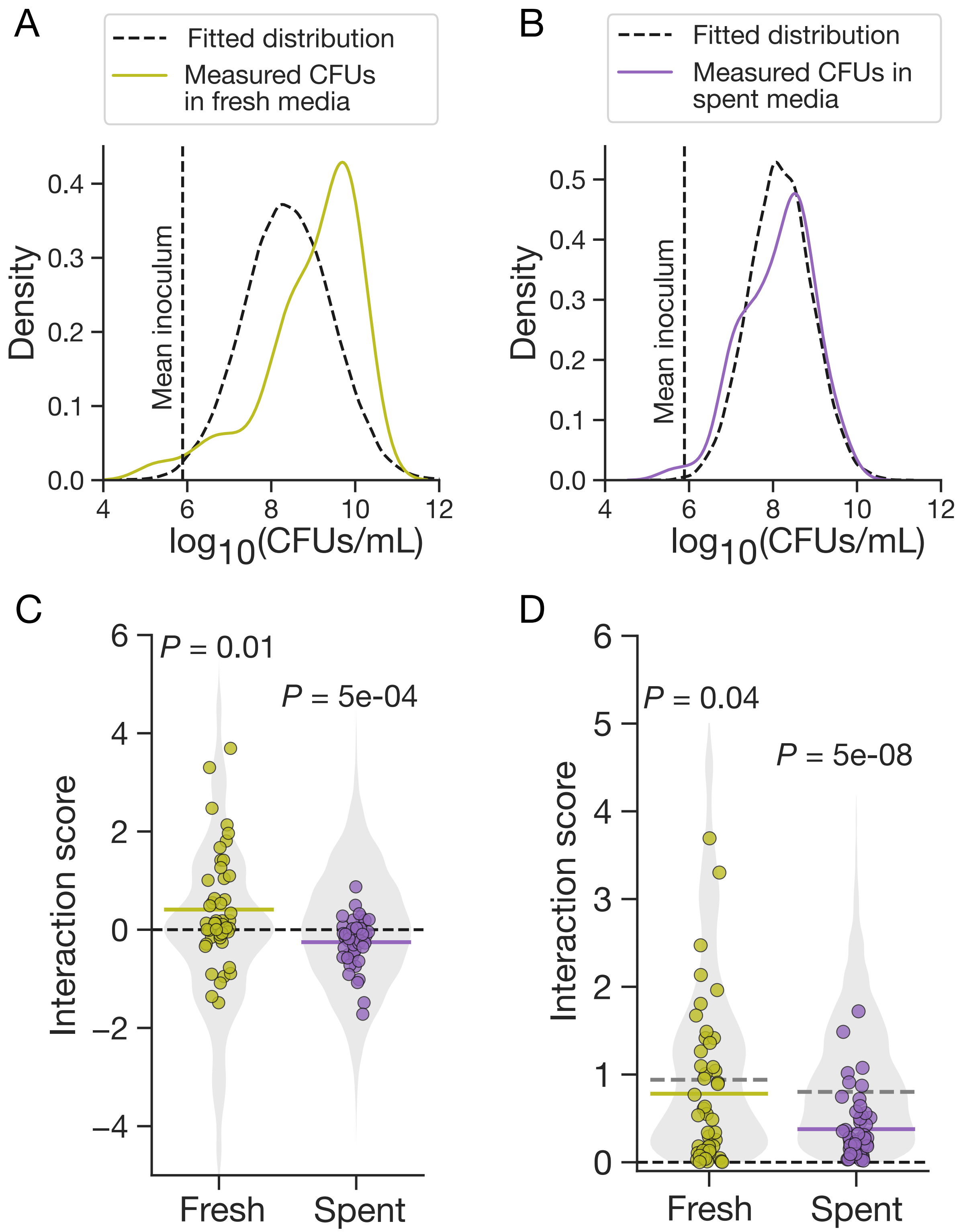

Fig. S7. Random models of interaction scores. A and B) Distributions of $\log_{10}$-transformed CFU values and lognormal distributions fitted to 5% and 95% percentiles of measured interactions, used for comparisons in Fig. 5. C) Interaction scores across fresh and spent media compared against null distributions from random models based on permutations of measured CFU values (Methods). Coloured horizontal bars indicate mean values. A two-sided one-sample Welch's *t*-test was used to assess the significance of the mean deviation from zero. D) Magnitude of interaction scores compared against null distributions from random models (as in C). Colored and gray horizontal bars represent the median absolute scores of the experimental data and the null models, respectively. A two-sided one-sample Wilcoxon signed-rank test was used to assess whether the observed median magnitude significantly deviated from the null expectation.

Table S1. The 20 amino acids used to supplement cultures for growth phenotyping.

**Amino acid**	**Source**
Alanine	Sigma, L-Alanine 05129-25G Lot # BCBW1873
Arginine	Fluka, L-Arginine 11009-500G, 334394/1 41797
Asparagine	Sigma, L-Asparagine, A0884-25G, Lot # SLCJ1974
Aspartate	Sigma-Aldrich, L-Aspartic acid potassium salt A6558-25G, Lot # WXBD5111V
Cysteine	Sigma, L-Cysteine C7352-100G, Lot # SLCJ3530
Glutamic acid	Sigma, L-Glutamic acid G8415-100G, Lot # SLCB8544
Glutamine	Sigma, L-Glutamine G3126-100G, Lot # SLBF5381V
Glycine	Biosolve, Glycine, Lot # 0010010402
Histidine	Sigma, L-Histidine 53319-25G, Lot # BCCB3337
Isoleucine	Sigma, L-isoleucine 58879-10G, Lot # BCCB6995
Leucine	Sigma, L-Leucine L8000-100G, Lot # BCCB8164
Lysine	Sigma-Aldrich, L-lysine monohydrochloride L5626-100G, Lot # SLBX8437
Methionine	Sigma, L-Methionine M9625-100G, Lot # 087KO236
Phenylalanine	Sigma-Aldrich, L-Phenylalanine P2126-100G, Lot #BCBC5774
Proline	Sigma, L-Proline C81709-25G, Lot #BCBZ5040
Serine	Sigma, L-Serine S4500-100G, Lot # 098K0161V
Threonine	Sigma-Aldrich, L-Threonine T8625-25G, Lot # SLBK2255V
Tryptophan	Sigma, L-Tryptophan T8941-25G, Lot # SLCH9077
Tyrosine	Fluka, L-Tyrosine 93830-100G, 1277054/20408005
Valine	Sigma-Aldrich, L-Valine V0500-25G, Lot # SLBH5511V

Table S2. Base aerobic medium for constraint-based analyses. Note that the carbon source is not listed here as it varies between conditions.

Exchange reaction	Lower bound (mmol/gDW/h)	Upper bound (mmol/gDW/h)
EX_nh4_e	-1000	1000
EX_pi_e	-1000	1000
EX_so4_e	-1000	1000
EX_h2o_e	-1000	1000
EX_h_e	-1000	1000
EX_o2_e	-1000	1000
EX_fe2_e	-1000	1000
EX_fe3_e	-1000	1000
EX_cobalt2_e	-1000	1000
EX_ca2_e	-1000	1000
EX_cl_e	-1000	1000
EX_cu2_e	-1000	1000
EX_k_e	-1000	1000
EX_mg2_e	-1000	1000
EX_mn2_e	-1000	1000
EX_zn2_e	-1000	1000
EX_mobd_e	-1000	1000
EX_ni2_e	-1000	1000
EX_na1_e	-1000	1000

Table S3. Carbon sources used for growth phenotyping.

Metabolite name	BiGG ID	Metabolite class	Source
Acetate	ac	Organic acid	Sigma-Aldrich, Sodium acetate S2889-1KG Lot #SLCC3512
Adenosine	adn	Nucleoside	Sigma, Adenosine 01890-25G Lot #BCBV4960
Arabinose	arab__D	Simple sugar	Fluka, D(-)-Arabinose 10850 414683/1 31001
Citrate	cit	Organic acid	Merck, Citric acid monohydrate C1.00244.1000
Cysteine	cys__L	Amino acid	Sigma, L-Cysteine C7352-100G Lot #SLCJ3530
Formate	for	Organic acid	Merck, Sodium formate GR 6443-500G
Fumarate	fum	Organic acid	Sigma-Aldrich, Fumaric acid 47910-100G, Lot # BCCB9913
Glucose	glc__D	Simple sugar	Applichem, D(+)-Glucose anhydrous, A0883, 1000 Lot #0U007223
Glutarate	glutar	Organic acid	Aldrich, Glutaric acid, G340-7 Lot # S44628-379
Glycerol	glyc	Alcohol	Applichem, Glycerol (pure), 141339.1212, Lot #0002188581
Glycine	gly	Amino acid	Biosolve, Glycine, Lot #0010010402
Inosine	ins	Nucleoside	Sigma, Inosine I4125-10G, lot #019K1124V
L-alanine	ala__L	Amino acid	Sigma, L-Alanine 05129-25G Lot #BCBW1873
L-arginine	arg__L	Amino acid	Fluka, L-Arginine 11009-500G, 334394/1 41797
L-aspartate	asp__L	Amino acid	Sigma-Aldrich, L-Aspartic acid potassium salt A6558-25G, Lot #WXBD5111V
L-glutamate	glu__L	Amino acid	Sigma, L-glutamate G8415-100G lot # SLCB8544
L-glutamine	gln__L	Amino acid	Sigma, L-Glutamine G3126-100G, Lot #SLBF5381V
L-histidine	his__L	Amino acid	Sigma, L-Histidine 53319-25G lot # BCCB3337
L-isoleucine	ile__L	Amino acid	Sigma, L-isoleucine 58879-10G lot #BCCB6995
L-leucine	leu__L	Amino acid	Sigma, L-Leucine L8000-100G, Lot #BCCB8164
Lactate	lac__L	Organic acid	Sigma-Aldrich, Sodium DL-lactate 71720-25G, Lot #BCCK4140
Malate	mal__L	Organic acid	Sigma-Aldrich, L-(-)-Malic acid, 112577-25G Lot #05122HE
Mannitol	mnl	Alcohol	Sigma-Aldrich, D-Mannitol, M4125-500G Lot # WXBC4630V
Mannose	man	Simple sugar	Roth, D (+)-Mannose 8887.2 Charge 219270993
myo-inositol	inost	Other	Fluka, myo-Inositol 57570 100g lot# 289364 290
Orotate	orot	Organic acid	Sigma, Orotic acid O2750-10G, 088K1235
Phenylalanine	phe__L	Amino acid	Sigma-Aldrich, L-Phenylalanine P2126-100G, Lot #BCBC5774
Proline	pro__L	Amino acid	Sigma, L-Proline C81709-25G, Lot #BCBZ5040
Pyruvate	pyr	Organic acid	Sigma-Aldrich, Sodium pyruvate P2256-25G, Lot #SLCP1313
Ribose	rib__D	Simple sugar	Sigma-Aldrich, D-(-)-Ribose W379301-100G Lot #088K0691V
Sucrose	sucr	Simple sugar	Sigma-Aldrich, Sucrose 84100-1KG, Lot #BCCJ6435
Uridine	uri	Nucleoside	Fluka, Uridine 94320-5G, 396594/1 12501
Xylose	xyl__D	Simple sugar	Applichem, D(+)-Xylose A2241,0500 lot # 1N014314

SI Dataset S1 (Dataset_S1.xlsx)

Dataset S1: Measured interaction scores in fresh and spent media.

SI Dataset S2 (Dataset_S2.xlsx)

Dataset S2: Summary of conditions for mono- and co-cultures in fresh and spent media.
